# TGF-β1 Decreases Microglia-Mediated Neuroinflammation and Lipid Droplet Accumulation in an In Vitro Stroke Model

**DOI:** 10.3390/ijms242417329

**Published:** 2023-12-10

**Authors:** Wenqiang Xin, Yongli Pan, Wei Wei, Stefan T. Gerner, Sabine Huber, Martin Juenemann, Marius Butz, Mathias Bähr, Hagen B. Huttner, Thorsten R. Doeppner

**Affiliations:** 1 Department of Neurology, University of Göttingen Medical School, 37075 Goettingen, Germany; wenqiang.xin@stud.uni-goettingen.de (W.X.); yongli.pan@stud.uni-goettingen.de (Y.P.); wei.wei01@stud.uni-goettingen.de (W.W.); mbaehr@gwdg.de (M.B.); 2 Department of Neurology, University of Giessen Medical School, 35392 Giessen, Germany; stefan.gerner@neuro.med.uni-giessen.de (S.T.G.); martin.juenemann@neuro.med.uni-giessen.de (M.J.); marius.butz@neuro.med.uni-giessen.de (M.B.); hagen.huttner@neuro.med.uni-giessen.de (H.B.H.); 3 Center for Mind, Brain and Behavior (CMBB), University of Marburg and Justus Liebig University, 35032 Giessen, Germany; 4 Heart and Brain Research Group, Kerckhoff Heart and Thorax Center, 61231 Bad Nauheim, Germany; 5 Department of Anatomy and Cell Biology, Medical University of Varna, 9238 Varna, Bulgaria; 6 Research Institute for Health Sciences and Technologies (SABITA), Medipol University, 100098 Istanbul, Turkey

**Keywords:** inflammation, lipid droplet, lipopolysaccharide, microglia, oxygen-glucose deprivation, stroke, TGF-β1

## Abstract

*H*ypoxia triggers reactive microglial inflammation and lipid droplet (LD) accumulation under stroke conditions, although the mutual interactions between these two processes are insufficiently understood. Hence, the involvement of transforming growth factor (TGF)-β1 in inflammation and LD accumulation in cultured microglia exposed to hypoxia were analyzed herein. Primary microglia were exposed to oxygen-glucose deprivation (OGD) injury and lipopolysaccharide (LPS) stimulation. For analyzing the role of TGF-β1 patterns under such conditions, a TGF-β1 siRNA and an exogenous recombinant TGF-β1 protein were employed. Further studies applied Triacsin C, an inhibitor of LD formation, in order to directly assess the impact of LD formation on the modulation of inflammation. To assess mutual microglia-to-neuron interactions, a co-culture model of these cells was established. Upon OGD exposure, microglial TGF-β1 levels were significantly increased, whereas LPS stimulation yielded decreased levels. Elevating TGF-β1 expression proved highly effective in suppressing inflammation and reducing LD accumulation in microglia exposed to LPS. Conversely, inhibition of TGF-β1 led to the promotion of microglial cell inflammation and an increase in LD accumulation in microglia exposed to OGD. Employing the LD formation inhibitor Triacsin C, in turn, polarized microglia towards an anti-inflammatory phenotype. Such modulation of both microglial TGF-β1 and LD levels significantly affected the resistance of co-cultured neurons. This study provides novel insights by demonstrating that TGF-β1 plays a protective role against microglia-mediated neuroinflammation through the suppression of LD accumulation. These findings offer a fresh perspective on stroke treatment, suggesting the potential of targeting this pathway for therapeutic interventions.

## 1. Introduction

Under physiological conditions, microglia, as resident immune cells of the central nervous system (CNS), are essential for preserving brain homeostasis [1]. Upon the occurrence of a stroke, these immune cells are activated in a dynamic process, providing both neurotoxic and neuroprotective effects. Intralesional microglia and newly recruited microglia assume an anti-inflammatory phenotype at the early stages of ischemia but gradually transform into a pro-inflammatory phenotype in lesion-neighboring areas [2]. These different biological properties of microglia exposed to stroke correlate with distinct phenotypes, as suggested by the pro-inflammatory M1 phenotype and the anti-inflammatory M2 phenotype [3]. Of note, such categorization between M1 and M2 microglia merely reflects two ends of a continuous spectrum. Nevertheless, the various microglial phenotypes are associated with different secretion patterns. M1-type microglia, for instance, secrete the pro-inflammatory cytokines tumor necrosis factor (TNF)-α, inducible nitric oxide synthase (iNOS), and interleukin (IL)-1β, whereas M2-type microglia rather display an anti-inflammatory cytokine panel, among which are IL-4, IL-10, and transforming growth factor (TGF)-β1 [4,5]. Controlling the shift of resident microglia from M1 towards M2 therefore implies an interesting therapeutic target for stroke treatment [6].

Lipid droplet (LD) biology of the brain has gained increasing interest in recent years [7,8,9]. These dynamic lipid storage organelles located in the cell cytoplasm significantly contribute to cellular lipid turnover and stress response by providing substrates for energy metabolism and biological membranes [10,11]. Under physiological conditions, however, only low amounts of LD are found in the brain [12], whereas aging or various noxious stimuli highly increase the LD accumulation of residing microglia [13]. Such LD-accumulating reactive microglia contribute to neurodegenerative disease progression, as indicated by the secretion of cytokines and reactive oxygen species. As such, secretion products of LD-enriched microglia have been recently implicated in the pathophysiology and development of neuroinflammatory disorders [8,14,15]. Suppressing LD accumulation in reactive microglia may therefore be an interesting target for maintaining cell homeostasis under conditions of stroke. The processes and metabolic disturbances that cause excessive LD accumulation in reactive microglia, however, are still not completely understood.

The TGF-β isoforms TGF-β1, TGF-β2, and TGF-β3 are pleiotropic cytokines critically involved in immune cell activation, inflammatory response, and post-injury repair. Microglia, astrocytes, endothelial cells, and the choroid plexus have been identified as the main sources of brain-derived TGF-β1 [16]. Using a preclinical stroke model, previous work from our group demonstrated that elevated TGF-β1 levels in microglia correlate with the extent of cell and tissue injury [17]. In that study, TGF-β1 was hypothesized to be a key mediator driving microglia towards an anti-inflammatory M2 activation state by applying an autofeedback loop. Although there are numerous studies investigating the anti-inflammatory effects of TGF-β1 on microglial cells [18,19], detailed mechanistic insight is still lacking. As a matter of fact, sustained suppressive effects of TGF-β1 on LD accumulation have never been reported. In the current study, we, therefore, addressed the role of TGF-β1 in the accumulation of LD in microglia using an in vitro stroke model and an LPS-induced inflammation model. We conclude this work by determining the putative effects of suppressed LD accumulation on the inflammatory profile of microglia.

## 2. Results

### 2.1. Accumulation of LD in Primary Microglia

**Figure 1:**
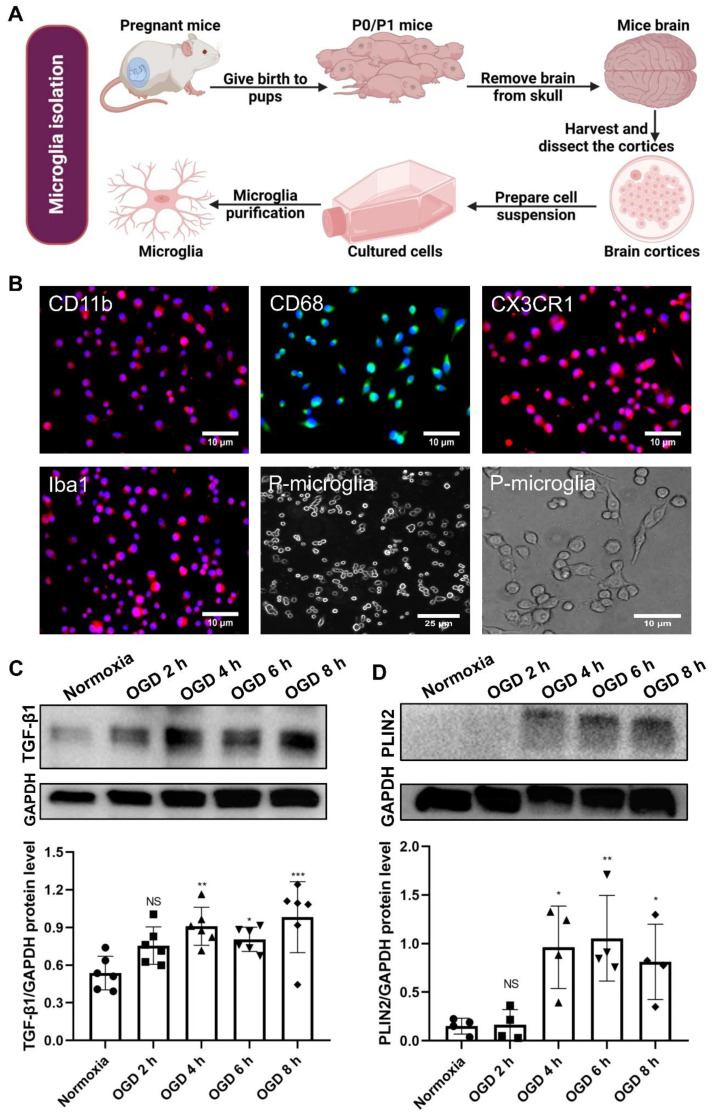
Characterization of primary microglia and the impact of hypoxia durations on TGF-β1 and LD-associated protein PLIN2 expression. (**A**) Schematic illustration of the culture of primary cortical microglia. Neonatal C57BL/6 mice were used to extract primary microglia, which were then cultured for 24 h prior to experimentation. (**B**) Immunofluorescence staining revealed the expression of CD11b, CD68, CX3CR1, and Iba1 in microglia. DAPI was employed as a counterstain for the cell nuclei. Additionally, light microscopy of primary microglia at p3 passage in culture (**B**) demonstrated a physiological and vital appearance, displaying processes and a ramified morphology. (**C**,**D**) The impact of OGD duration followed by 24 h of reoxygenation on TGF-β1 and LD-associated protein PLIN2 expression was measured by western blot (*n =* 4–6). * *p* < 0.05; ** *p* < 0.01; *** *p* < 0.001. LD, lipid droplet(s); P-microglia, primary microglia; NS, not statistically significant; TGF-β1, transforming growth factor-β1; OGD, oxygen-glucose deprivation; PLIN2, perilipin2.

Cortices of newborn C57BL/6 mice were used to isolate primary microglial cell cultures as previously described [20], with a few minor modifications (Figure 1A). Before primary microglia were used for the experimental protocol, immunofluorescence techniques were used to analyze the displayed “typical” expression patterns known for microglia, such as CD11b, CD68, CX3CR1, and Iba1 (Figure 1B). Indeed, such analysis revealed a typical microglial expression pattern together with a microglial morphology as assessed using a light microscope (Figure 1B). Thereafter, the behavior of these cultured microglia under in vitro stroke conditions was assessed, finding the optimal parameters for the OGD model. When primary microglia were subjected to different durations of hypoxia followed by a 24-h reoxygenation period, after 4 h, 6 h, and 8 h of hypoxia followed by 24 h of reoxygenation, TGF-β1 and Perilipin-2 (PLIN2), a prominent LD-associated protein, were significantly increased (Figure 1C,D).

**Figure 2:**
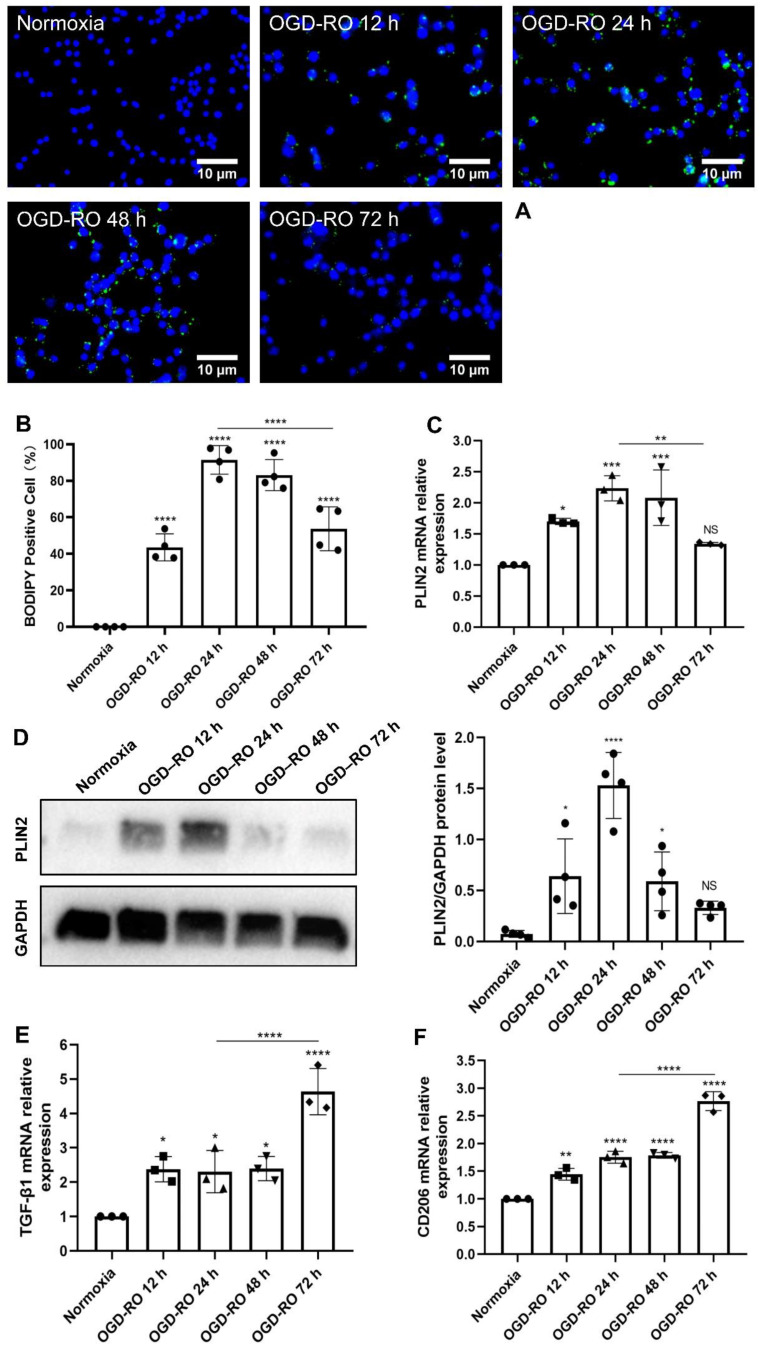
The dynamic changes of LD formation, TGF-β1 expression, and M2 microglia polarization following OGD**.** (**A**) Dynamic evolution of LD immunostaining (BODIPY staining) in primary microglia exposed to 4 h of OGD followed by either 12 h, 24 h, 48 h, or 72 h of RO. (**B**) Quantitative analysis of BODIPY-positive cells in primary microglia of different RO groups from (**A**) with *n* = 4. (**C**) RT-qPCR was employed to detect the LD-associated gene PLIN2 on the mRNA level (*n* = 3). (**D**) The dynamic PLIN2 protein expression in primary microglia exposed to 4 h of OGD followed by different periods of RO was measured by western blot (*n* = 4). (**E**,**F**) RT-qPCR analysis assay of TGF-β1 mRNA and M2 microglia polarization marker CD206 mRNA in primary microglia in 5 groups: normoxia, 4 h of OGD followed by 12-h RO, 4 h of OGD followed by 24-h RO, 4 h of OGD followed by 48-h RO, and 4 h of OGD followed by 72-h RO (*n* = 3). * *p* < 0.05; ** *p* < 0.01; *** *p* < 0.001; **** *p* < 0.0001. LD, lipid droplet(s); NS, not statistically significant; OGD, oxygen-glucose deprivation; RO, reoxygenation; RT-qPCR, quantitative real-time PCR analysis; TGF-β1, transforming growth factor-β1; PLIN2, perilipin2.

Setting different periods of reoxygenation, i.e., 12 h, 24 h, 48 h, and 72 h after the hypoxia period of 4 h, an immunofluorescence technique was employed to assess LD accumulation. As indicated in Figure 2A,B, LD accumulation was significantly increased in all aforementioned periods of reoxygenation. Using RT-qPCR and Western blot technology (Figure 2C,D), compared with the normoxic group, PLIN2 gene and protein levels were increased in groups of 12 h, 24 h, and 48 h of reoxygenation. However, no significant difference was identified in the group after 72 h of reoxygenation. Of note, in the group of 72 h, LD accumulation and the PLIN2 gene yielded a downward trend when compared with the group of 24 h. On the contrary, TGF-β1 mRNA was significantly increased at all reoxygenation periods compared with those in the normoxia group (Figure 2E). Not only did the expression level of TGF-β1 positively correlate with the duration of reoxygenation, but it was also positively correlated with M2 signature genes (CD206, Cluster of Differentiation 206) (Figure 2F). We hypothesized that the gradual elevation of TGF-β1 levels over time could be responsible for the successive anti-inflammatory upregulation of microglia under OGD conditions. For the remainder of the study, 4 h of OGD followed by 24 h of reoxygenation served as the experimental setting for the hypoxic preconditioning of cultured microglia.

### 2.2. TGF-β1 Regulates OGD-Induced LD Accumulation

**Figure 3:**
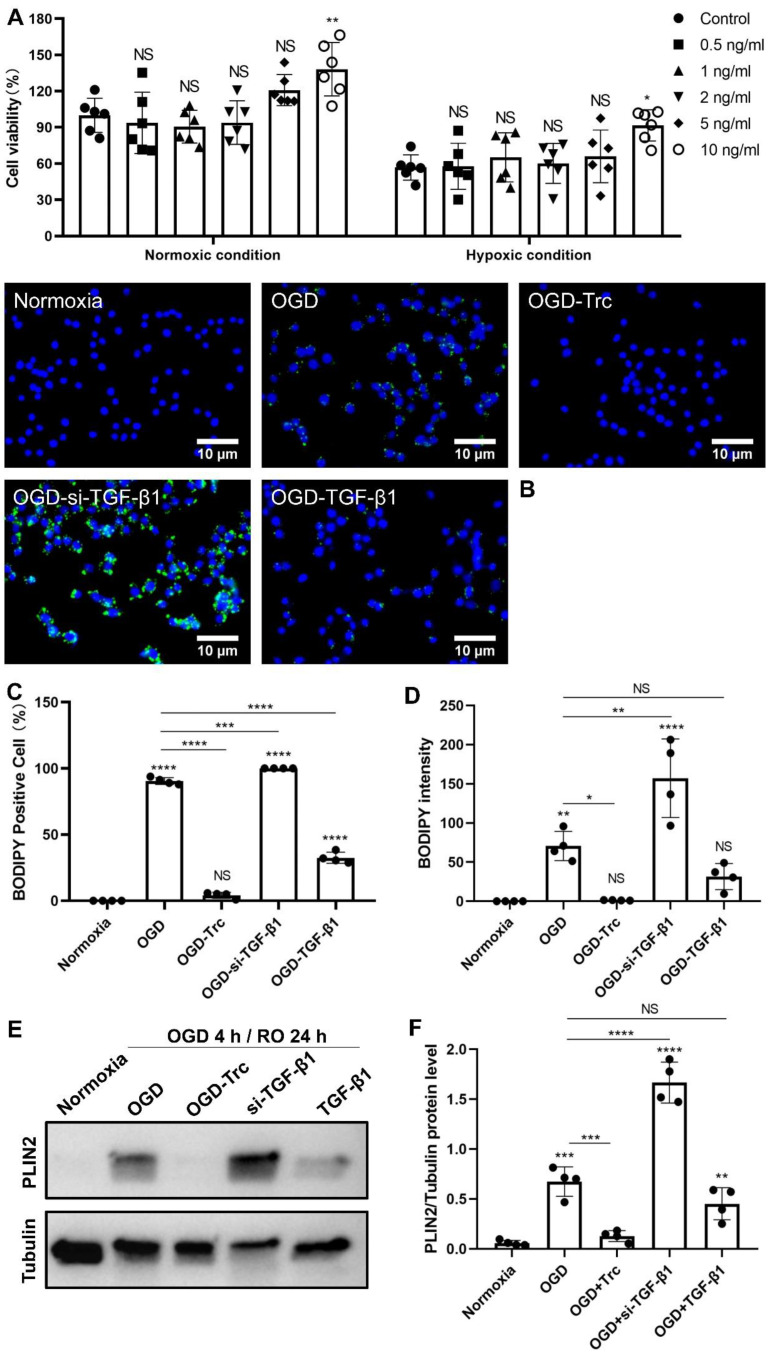
TGF-β1 regulates OGD-induced LD accumulation and cell survival of primary microglia in a concentration-dependent fashion. MTT was used to test the impact of different concentrations of exogenous TGF-β1 on microglial cell viability exposed to normoxia or to 4 h of OGD followed by 24 h of reoxygenation (**A**). Cells incubated under standard cell culture conditions (‘Normoxia’) were defined as 100% cell survival (*n* = 6). (**B**) LD immunostaining (BODIPY staining) in primary microglia exposed to different treatment paradigms. (**C**,**D**) Statistical plots of BODIPY-positive cells and BODIPY intensity in the primary microglia of different treatment groups (*n* = 4). (**E**,**F**) Western blot analysis showing PLIN2 protein expression in primary microglia after OGD in untreated cells or cells treated with Triacsin C, TGF-β1, and TGF-β1 siRNA (*n* = 4). * *p* < 0.05; ** *p* < 0.01; *** *p* < 0.001; **** *p* < 0.0001. LD, lipid droplet(s); MTT, thiazolyl blue tetrazolium bromide; NS, not statistically significant; OGD, oxygen-glucose deprivation; PLIN2, perilipin2; si-TGF-β1, TGF-β1 siRNA; TGF-β1, transforming growth factor-β1; Trc, Triacsin C.

To reveal the optimal TGF-β1 concentration for microglial cell viability, TGF-β1 in concentrations of 0.5 ng/mL, 1 ng/mL, 2 ng/mL, 5 ng/mL, and 10 ng/mL was used in primary microglia exposed to normoxia and OGD (Figure 3A). TGF-β1 significantly increased microglia viability in a concentration-dependent manner. The protective impact of TGF-β1, however, was not significant when TGF-β1 was applied at a concentration of 0.5–5 ng/mL, suggesting that a low concentration of TGF-β1 may be insufficient for enhancing the resistance of primary microglial cells under such conditions. Rather, a high concentration of TGF-β1 at 10 ng/mL confers higher cell viability in normoxic microglia and significant protection against OGD-induced cell death in primary microglia.

To further measure the relationship between TGF-β1 levels and LD accumulation, a recombinant mouse TGF-β1 protein and a TGF-β1 siRNA were used in primary microglia exposed to OGD. Using immunofluorescence measurement, inhibition of TGF-β1 using TGF-β1 siRNA significantly increased the percentage of BODIPY-positive cells (Figure 3B,C). On the contrary, the increase in TGF-β1 levels using exogenous recombinant mouse TGF-β1 protein significantly decreased the percentage of BODIPY-positive cells (Figure 3B,C). When considering the analysis of BODIPY intensity, the reduction in TGF-β1 levels using an siRNA significantly increased the density of BODIPY staining, while an increase in TGF-β1 levels did not show a reversed outcome (Figure 3D). Western blot analyses on the PLIN2 protein level confirmed the immunohistochemical data regarding the BODIPY intensity (Figure 3E,F). We used Triacsin C, an inhibitor of long-chain acyl-CoA synthetase, to confirm the identity of these BODIPY-labeled LD. Triacsin C hinders the production of LD by inhibiting the de novo synthesis of glycerolipids. In fact, Triacsin C treatment reversed the OGD-induced rise in BODIPY density, BODIPY cell percentage, and PLIN2 protein level.

### 2.3. TGF-β1 Can Regulate LPS-Induced LD Accumulation

**Figure 4:**
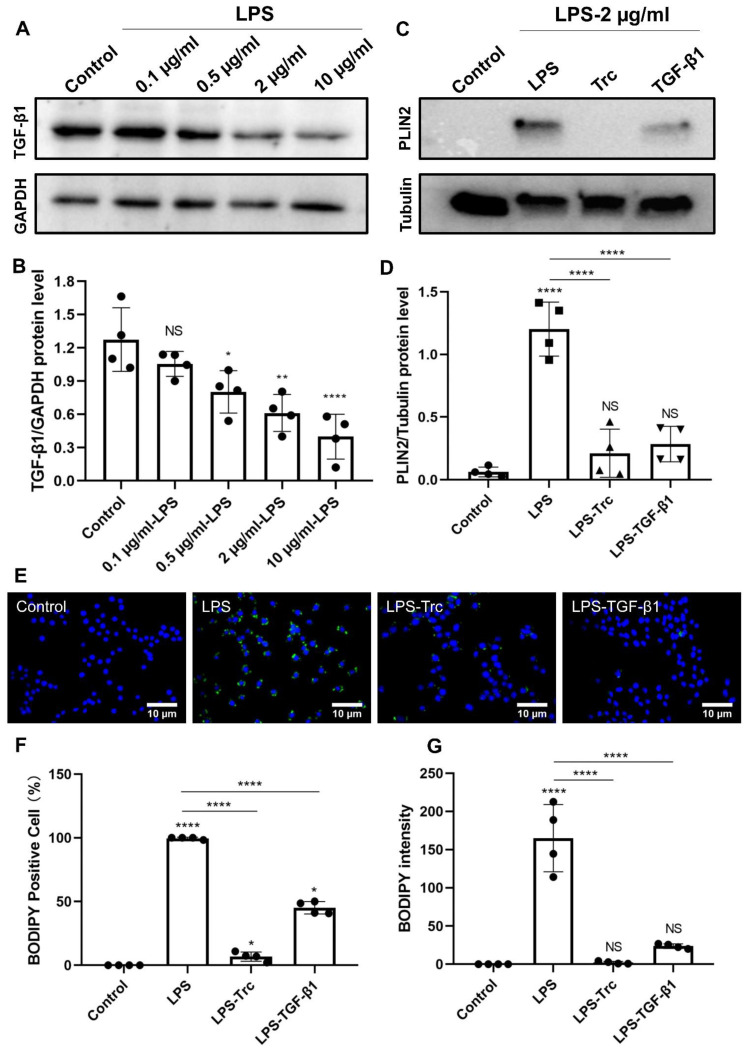
TGF-β1 regulates LPS-induced LD accumulation in primary microglia. (**A**,**B**) The influence of various concentrations of LPS (0.1 μg/mL, 0.5 μg/mL, 2 μg/mL, and 10 μg/mL) on the protein level of TGF-β1 was assessed through western blot analysis. Primary microglia were exposed to different doses of LPS for 24 h, resulting in a dose-dependent effect on the TGF-β1 level (*n* = 4). (**C**,**D**) Western blot analysis shown for the expression of PLIN2 protein in primary microglia under different conditions, including untreated cells, LPS-treated cells, LPS-treated cells with Triacsin C, and LPS-treated cells with TGF-β1 (*n* = 4). (**E**) LD immunostaining (BODIPY staining) in primary microglia exposed to different treatments. (**F**,**G**) Statistical plots of BODIPY positive cells and BODIPY intensity in the primary microglia of different treatment groups, including untreated cells, LPS-treated cells, LPS-treated cells with Triacsin C, and LPS-treated cells with TGF-β1 (*n* = 4). * *p* < 0.05; ** *p* < 0.01; **** *p* < 0.0001. LD, lipid droplet(s); LPS, lipopolysaccharide; NS, not statistically significant; PLIN2, perilipin2; TGF-β1, transforming growth factor-β1; Trc, Triacsin C.

Given the above inconsistent analysis of BODIPY density and percentage of BODIPY-positive cells in the in vitro OGD stroke model, further analysis of TGF-β1 on LD accumulation in primary microglia exposed to LPS was conducted. The concentration of LPS was selected mainly based on its effect on the TGF-β1 level. Exposure of primary microglia to different doses of LPS for 24 h revealed a dose-dependent extent of the TGF-β1 level (Figure 4A,B). LPS at 2 µg/mL and 10 µg/mL can significantly reduce the TGF-β1 protein level by a rate of about 50%. Further experiments were performed with an LPS concentration of 2 µg/mL, which did not yield a significant cell injury. When primary microglial cells were given recombinant mouse TGF-β1 protein, however, the PLIN2 protein level was decreased (Figure 4C,D), suggesting a suppressive action for TGF-β1 on LD accumulation upon LPS stimulation. Thereafter, immunofluorescence measurements on BODIPY density and percentage of BODIPY-positive cells confirmed the western blot data (Figure 4E–G). In addition, treatment with Triacsin C abolished the LPS-induced increase in BODIPY density, percentage of BODIPY-positive cells, and PLIN2 protein level.

### 2.4. TGF-β1 Modulates Microglia-Mediated Neuroinflammation

**Figure 5:**
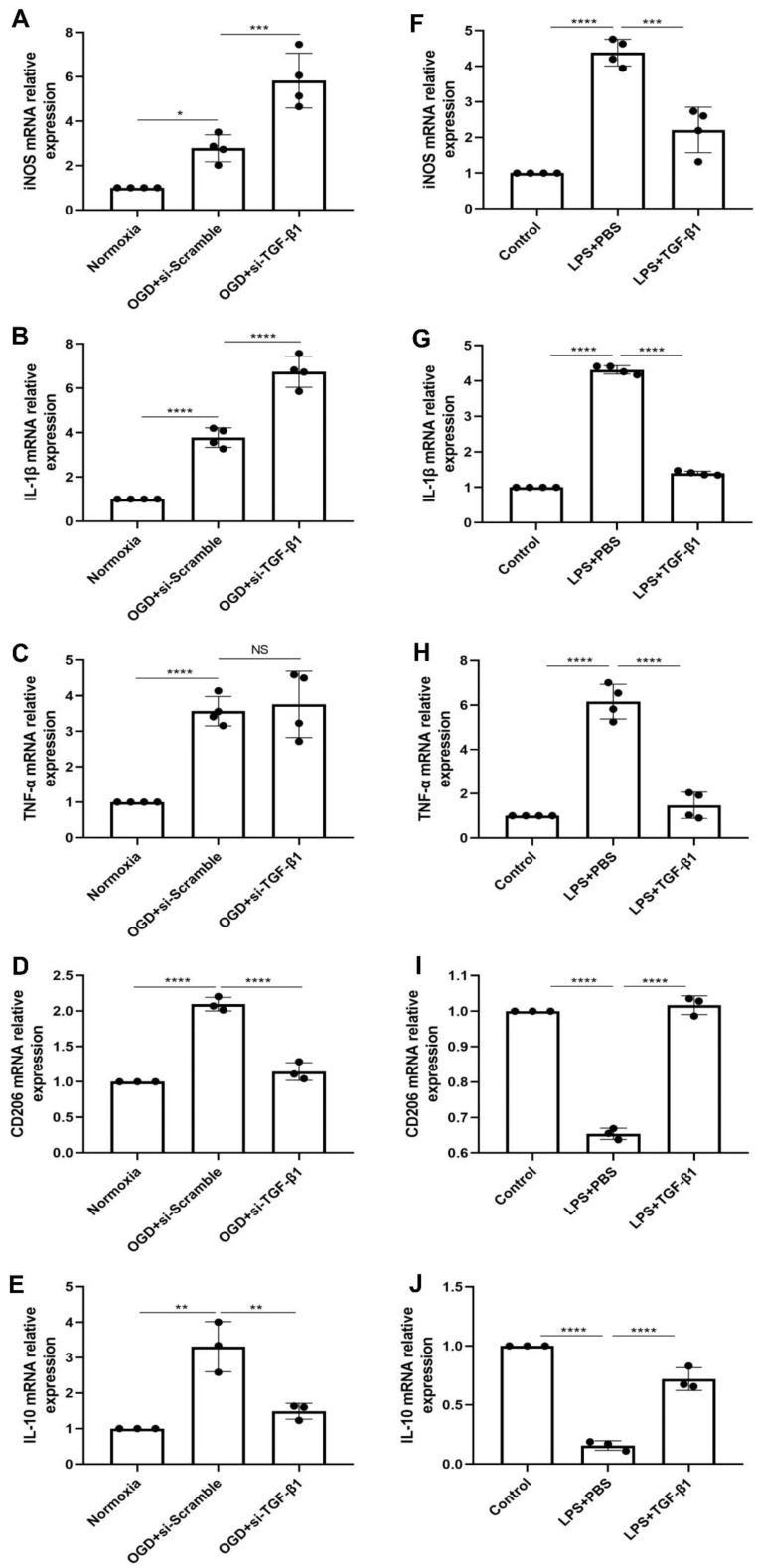
TGF-β1 regulates microglia-mediated neuroinflammation. (**A**–**C**) RT-qPCR analysis assay of M1 microglia polarization marker iNOS, IL-1β, and TNF-α mRNA in primary microglia in three groups: normoxia, 4 h of OGD followed by 24-h reoxygenation with siRNA scramble treatment, and 4 h of OGD followed by 24-h reoxygenation with TGF-β1 siRNA treatment (*n* = 4). (**D**,**E**) RT-qPCR analysis assay of M2 microglia polarization marker CD206 and IL-10 mRNA in primary microglia after OGD in cells treated with siRNA scramble or cells treated with TGF-β1 siRNA (*n* = 3). (**F**–**H**) RT-qPCR analysis assay of M1 microglia polarization marker iNOS, IL-1β, and TNF-α mRNA in primary microglia in three groups: control, 24 h of LPS with PBS, and 24 h of LPS with TGF-β1 treatment (*n* = 4). (**I**,**J**) RT-qPCR analysis assay of M2 microglia polarization marker CD206 and IL-10 mRNA in primary microglia under LPS conditions (*n* = 3). * *p* < 0.05; ** *p* < 0.01; *** *p* < 0.001; **** *p* < 0.0001. IL, interleukin; iNOS, inducible nitric oxide synthase; LPS, lipopolysaccharide; NS, not statistically significant; OGD, oxygen-glucose deprivation; RT-qPCR, quantitative real-time PCR analysis; si-TGF-β1, TGF-β1 siRNA; TGF-β1, transforming growth factor-β1; TNF-α, tumor necrosis factor-α.

To directly address whether or not TGF-β1 exerted its protective functions on microglia-mediated neuroinflammation, we employed OGD and LPS in primary cultured microglia to induce inflammation in vitro, as previously described [21,22]. In the case of OGD, the TGF-β1 level was elevated, thus the effect of inhibition of TGF-β1 using siRNA in primary microglia exposed to OGD was examined (Figure 5A–E). Interestingly, incubation of primary microglia exposed to OGD with TGF-β1 siRNA shifted microglial cells towards an M1 polarization phenotype even further than OGD with siRNA scramble, as indicated by RT-qPCR data on mRNA levels of iNOS and IL-1β. M2 polarization, on the contrary, was diminished due to the application of TGF-β1 siRNA, as shown by reduced levels of CD206 and IL-10, which originate from M2 phenotype microglia. Interesting correlations were found between the application of TGF-β1 siRNA and decreased cell viability as well (Figure S1A). To further determine whether inflammation can be effectively inhibited by restoring the down-regulated TGF-β1 level caused by LPS, we compared the mRNA expressions of M1 (iNOS, IL-1β, and TNF-α) and M2 (CD206 and IL-10) microglia signature genes among the control, LPS+PBS, and LPS + TGF-β1 treatment groups. As expected, LPS stimulation caused an increase of iNOS, IL-1β, and TNF-α and a striking decrease of CD206 and IL-10. Treatment of TGF-β1, on the contrary, reversed the above changes in LPS-stimulated microglia (Figure 5F–J).

### 2.5. Inhibition of LD Formation Reduces Microglia-Mediated Neuroinflammation

**Figure 6:**
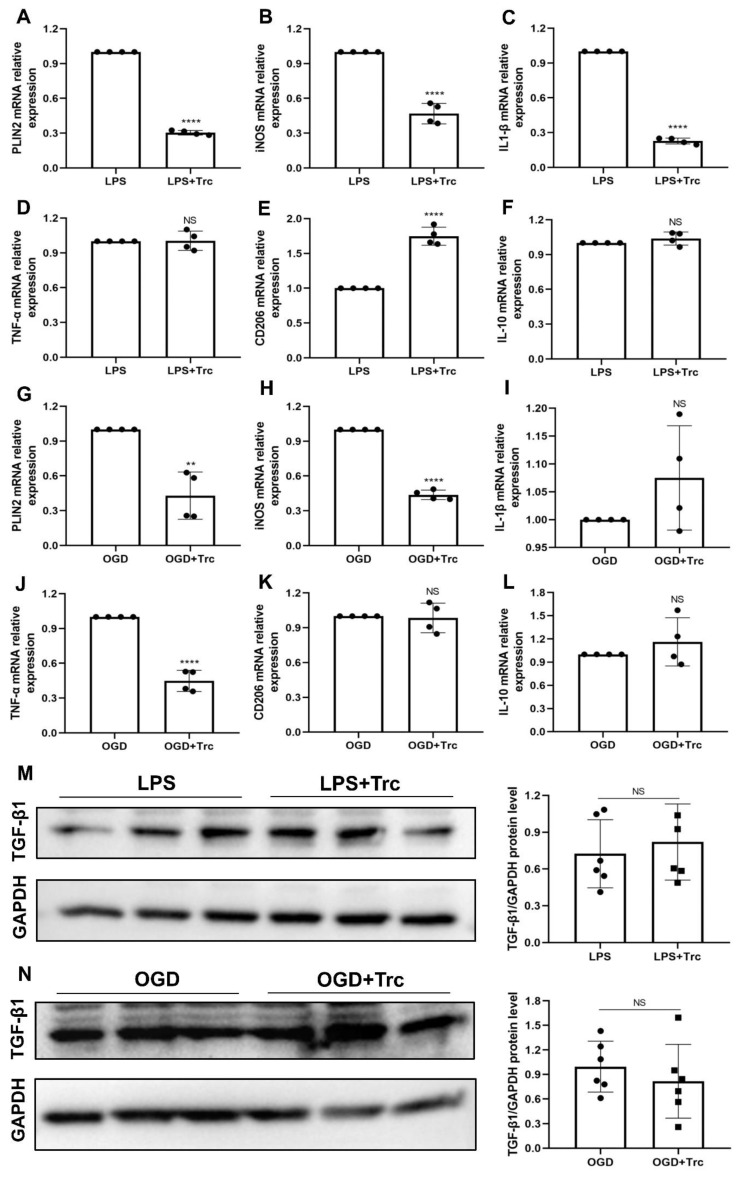
Inhibition of LD formation with Triacsin C sufficiently reduces microglia-mediated neuroinflammation under LPS and OGD conditions. (**A**) RT-qPCR analysis assay shows that Triacsin C suppresses the LD-associated gene PLIN2 mRNA expression in LPS-treated cells (*n* = 4). (**B**–**D**) RT-qPCR analysis assay of the pro-inflammatory cytokines iNOS, IL-1β, and TNF-α mRNA in primary microglia in two groups: cells received 24 h of LPS with PBS or 24 h of LPS with Trc treatment (*n* = 4). (**E**,**F**) RT-qPCR analysis assay of anti-inflammatory cytokines CD206 and IL-10 mRNA in primary microglia in two groups: cells received 24 h of LPS with PBS or 24 h of LPS with Trc treatment (*n* = 4). (**G**) RT-qPCR analysis assay showing Trc suppresses the LD-associated gene PLIN2 mRNA expression in OGD-treated cells (*n* = 4). (**H**–**J**) RT-qPCR analysis assay of pro-inflammatory cytokines iNOS, IL-1β, and TNF-α mRNA in primary microglia in two groups: cells received 4 h of OGD followed by 24-h reoxygenation with Trc treatment or not (*n* = 4). (**K**,**L**) RT-qPCR analysis assay of anti-inflammatory cytokines CD206 and IL-10 mRNA in primary microglia treated with 4 h of OGD followed by 24-h reoxygenation or 4 h of OGD followed by 24-h reoxygenation with Trc treatment (*n* = 4). (**M**,**N**) Western blot analysis for the depiction of TGF-β1 expression in primary microglia after Trc treatment under different conditions, including LPS-treated cells and OGD-treated cells (*n* = 6). ** *p* < 0.01; **** *p* < 0.0001. IL, interleukin; iNOS, inducible nitric oxide synthase; LD, lipid droplet(s); LPS, lipopolysaccharide. NS, not statistically significant; OGD, oxygen-glucose deprivation; PLIN2, perilipin2; RT-qPCR, quantitative real-time PCR analysis; TNF-α, tumor necrosis factor-α; Trc, Triacsin C; TGF-β1, transforming growth factor-β1.

LD-accumulating microglia represent a dysfunctional and pro-inflammatory state [8]. These cells are defective in phagocytosis, produce high levels of reactive oxygen species, and secrete pro-inflammatory cytokines [8]. Yet, little is known about the pharmacological role of LD formation with regard to suppressing microglia-mediated neuroinflammation. Hence, we treated primary microglia exposed to either OGD or LPS with Triacsin C, an inhibitor of LD formation (Figure S2) [8]. Upon LPS stimulation, pharmacological inhibition of LD formation due to Triacsin C prevented pro-inflammatory cytokine (iNOS and IL-1β) and promoted anti-inflammatory cytokines (CD206) generation, supporting the idea that LD may have a causal role in LPS-induced inflammation (Figure 6A–F). Likewise, inhibition of LD formation under OGD conditions reduced pro-inflammatory cytokine (iNOS and TNF-α) expression patterns (Figure 6G–L). Interestingly, no significant difference was identified with regard to anti-inflammatory cytokine expression patterns under such conditions. The anti-inflammatory effects observed through the inhibition of LD accumulation using Triacsin C, however, appear to be (in part) independent of an elevation of TGF-β1 levels (Figure 6M,N).

### 2.6. TGF-β1 Plays an Important Role in Primary Neuron Viability Exposed to Hypoxia

**Figure 7:**
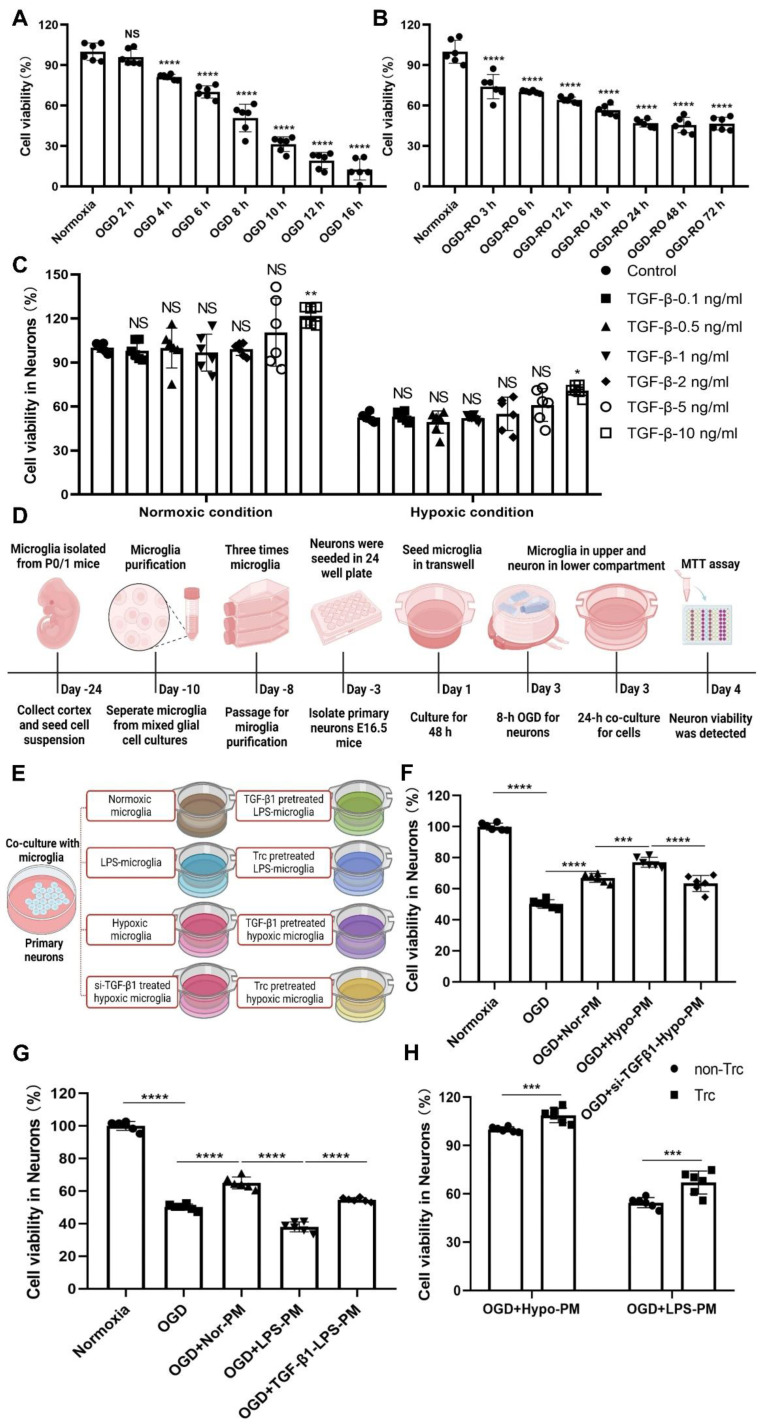
TGF-β1 plays an important role in neuronal cell viability under hypoxic conditions. (**A**) MTT (thiazolyl blue tetrazolium bromide) was used to test the cell viability of primary neurons exposed to 2 h, 4 h, 6 h, 8 h, 10 h, 12 h, and 16 h of OGD followed by 24-h reoxygenation (*n* = 6). (**B**) MTT was used to test the cell viability exposed to 8 h of OGD followed by 3 h, 6 h, 12 h, 18 h, 24 h, 48 h, or 72 h of reoxygenation. Cells incubated under standard cell culture conditions (‘Normoxia’) were defined as 100% cell survival (*n* = 6). (**C**) To test the effect of TGF-β1 on primary neurons under both normoxia and OGD conditions (OGD 8 h/RO 24 h), TGF-β1 was applied at concentrations of 0.1 ng/mL, 0.5 ng/mL, 1 ng/mL, 2 ng/mL, 5 ng/mL, and 10 ng/mL (*n* = 6). (**D**) Experimental paradigm summarizing the in vitro co-culture model of primary microglia and primary neurons. (**E**) The establishment of a co-culture system to facilitate microglia-neuron communication involved the seeding of microglia in the upper compartment and the seeding of primary neurons in the lower compartment. (**F**–**H**) An alteration of TGF-β1 levels and LD amount in modulating microglia-to-neuron in terms of primary neuronal cell viability (*n* = 6). * *p* < 0.05; ** *p* < 0.01; *** *p* < 0.001; **** *p* < 0.0001. Hypo, hypoxic; LD, lipid droplet(s); LPS, lipopolysaccharide; Nor, normoxic; NS, not statistically significant; OGD, oxygen-glucose deprivation; PM, primary microglia; RO, reoxygenation; si-TGF-β1, TGF-β1 siRNA; Trc, Triacsin C; TGF-β1, transforming growth factor-β1.

The influence of TGF-β1 on neuronal cell viability was assessed using primary cortical neurons in an OGD model. Exposure of cultured neurons to various durations of hypoxia (2 h, 4 h, 6 h, 8 h, 10 h, 12 h, or 16 h) followed by 24 h of reoxygenation showed that 8 h of hypoxia resulted in a moderate cell death rate of approximately 50% compared to normoxic neurons (Figure 7A). Varying reoxygenation periods (3 h, 6 h, 12 h, 18 h, 24 h, 48 h, and 72 h after 8 h of hypoxia) demonstrated that neuronal cell viability reached its minimum plateau phase after 24 h of reoxygenation (Figure 7B). Therefore, hypoxic preconditioning of primary neurons involved 8 h of OGD followed by 24 h of reoxygenation.

To examine the effect of TGF-β1 on OGD-induced neuronal cell injury, TGF-β1 at concentrations of 0.1 ng/mL, 0.5 ng/mL, 1 ng/mL, 2 ng/mL, 5 ng/mL, and 10 ng/mL was incubated with hypoxic neurons. Interestingly, the protective impact of TGF-β1 was not significant at concentrations of 0.1–5 ng/mL. However, a high concentration of 10 ng/mL conferred protection in hypoxic neurons (Figure 7C). This observation was confirmed when TGF-β1 at the same concentrations was incubated with normoxic neurons, yielding similar results as in the hypoxic condition (Figure 7C).

In a co-culture model, we further investigated whether or not microglia with different TGF-β1 levels exert distinct effects on OGD-induced neuronal cell damage. Primary microglia were isolated, purified, and subjected to various treatment paradigms (normoxia, 4 h of hypoxia with subsequent 24 h of reoxygenation, and 24 h of LPS). Simultaneously, primary neurons were seeded in 24-well plates, and the inserts with primary microglia were placed on the plates with primary neurons (Figure 7D,E). As illustrated in Figure 7F,G, primary neurons co-cultured with normoxic microglial cells exhibited a higher level of cell viability compared to neurons exposed to 8 h of OGD followed by 24 h of reoxygenation alone. Co-culturing with hypoxia-preconditioned microglia further enhanced neuronal survival. Additionally, a significant decrease in cell viability was observed in co-cultures with TGF-β1 siRNA-pretreated hypoxic microglia compared to the co-culture with hypoxic microglia alone. Co-culture of primary neurons with LPS-pretreated microglia resulted in decreased neuron survival, an effect reversed when primary neurons were simultaneously treated with both LPS-pretreated microglia and TGF-β1. Importantly, inhibition of LD formation due to Triacsin C treatment in combination with either OGD-preconditioned or LPS-preconditioned microglia significantly increased primary neuron viability under hypoxic conditions (Figure 7H). These results indicate that microglia indeed influence the cell viability of primary neurons. Moreover, alterations in TGF-β1 and LD levels appear to play a role in mediating the communication between microglia and neurons, thus affecting cell viability.

## 3. Discussion

Ischemic stroke affects both neurons and glial cells. The latter, however, are not only supporting cells for neurons but are also actively involved in brain remodeling, establishing a solid foundation for successful neurological rehabilitation [23]. Under physiological circumstances, microglia act as innate immunocompetent glia that diligently surveil their cerebral microenvironment [6]. Upon stroke induction, microglia take over either pro-inflammatory (M1-like phenotype) or anti-inflammatory (M2-like phenotype) roles, depending on temporal and spatial resolution patterns [4,24]. Of note, both M1-like and M2-such as microglia only represent two extremes of a dynamic spectrum, where microglia change their phenotype in response to their cerebral environment [25]. Such a change of phenotype involves the secretion of cytokines, including the TGF-β family [26,27]. Among the latter, the role of anti-inflammatory TGF-β1 has been studied in various tissues and organs, including the brain, where it exerts neuroprotective effects [28,29,30,31], although the precise mechanisms are not known. In the current study, we demonstrate that TGF-β1 plays a pivotal role in modulating microglial-induced inflammation during hypoxic conditions. To a certain extent, this modulation may occur through the inhibition of LD accumulation in microglial cells.

Noxious stimuli such as OGD or LPS yield increased LD formation in primary microglia, as indicated by our in vitro data. Application of exogenous TGF-β1, however, reverses this effect. These observations are supported by further in vivo studies performed in mice, showing that LPS administration is associated with an elevation in the number of LD within leukocytes [32]. Such LD have been associated with the innate immune response and have been described as “inflammatory organelles” that can serve as crucial indicators of cellular activation. Whereas LPS significantly enhances the accumulation of LD, it also significantly diminishes the expression level of TGF-β1 [33]. Hence, primary microglia, as used in our model, displayed a significant increase in LD accumulation when subjected to OGD. However, a time-dependent study of LD during a 72-h reoxygenation period following OGD stimulation demonstrated that the initial increase in LD count was not sustaining. After peaking at 24 h of reoxygenation treatment, the number of LD declined. This decrease could potentially be attributed to the continuously elevated level of TGF-β1, which acts in an autocrine manner. When we inhibited the level of TGF-β1 at the peak of the LD increase, we observed a notable and significant augmentation in the number of LD. This discovery robustly reinforces the idea that alterations in TGF-β1 levels can influence the accumulation of LD in microglial cells, either enhancing or diminishing the process. Indeed, exogenous recombinant mouse TGF-β1 protein applied to the cell culture yielded a significant reduction in the number of LD under OGD conditions. Other published work suggests that LD accumulation in microglia is dependent on triglyceride biosynthesis [15]. Hence, additional experiments focused on the inhibition of LD induction under both LPS and OGD settings by using Triacsin C, an inhibitor of long-chain acyl-CoA synthetase activity that is involved in triglyceride synthesis. As a matter of fact, LD accumulation was significantly reduced due to Triacsin C under such conditions.

The findings of the present work indicate that microglial TGF-β1 levels increase in response to OGD stimulation, whereas LPS stimulation yields decreased TGF-β1 levels. The differing changes in TGF-β1 expression observed in microglia under the influence of these two stimuli may indicate distinct inflammatory processes triggered by each stimulus. Inhibition of the TGF-β1 signaling pathway results in an augmented inflammatory response caused by OGD, indicating a dampening effect of this signaling pathway on inflammation under conditions of hypoxia. The administration of exogenous TGF-β1 increases the expression level of anti-inflammatory cytokines even further while reducing the release of pro-inflammatory cytokines when compared to primary microglial cells treated with LPS only. This is in line with previous published work demonstrating that expression levels of IL-1β, IL-6, and TNF-α are decreased upon an increase of TGF-β1 in microglial BV2 cells treated with nanoparticulate titanium dioxide [34].

Inflammation has been frequently associated with the development of LD [35]. This connection has been observed through ex vivo brain imaging as well as in vitro studies focusing on LD [35]. LPS, a bacterial endotoxin, acts as a natural ligand for TLR4 and serves as a proinflammatory stimulus for microglia activation [8,15]. In both in vitro and in vivo culture settings, LPS has been shown to induce an accumulation of LD in microglia by increasing the number and size of LD [8,15]. In LPS-activated microglia, LDs are believed to serve as platforms to produce inflammatory signaling molecules, specifically eicosanoids such as arachidonic acid [15]. A separate investigation revealed that the exposure of isolated primary astrocytes to palmitate results in elevated levels of inflammatory markers [36]. Additionally, staining with Oil Red O, a dye soluble in fats, along with the transcription of PLIN2, was increased. Interestingly, when microglia were treated with conditioned media derived from lipid-loaded astrocytes, enhanced chemotaxis of microglia through the CCR2-MCP1 mechanism was observed [36]. These findings indicate that LD loaded in astrocytes may serve as a signal to microglia, leading to the amplification of the inflammatory response. The increased presence of LD in the CNS can thus be considered a potential indication of a proinflammatory state [15], similar to the proposed indication in cancer cells [35]. Nonetheless, it remains to be determined whether or not LD serves as a consequence of inflammation, a cause of inflammation, or if both factors mutually affect each other.

In our work, using an in vitro inflammation model where primary microglia are exposed to OGD or LPS and subsequently treated with Triacsin C yielded an anti-inflammatory effect. This experimental approach rather suggests that the suppression of LD accumulation results in an anti-inflammatory effect and not vice versa. Nevertheless, a possible impact of elevated inflammation initially triggering LD formation and subsequent LD-mediated secretion of pro-inflammatory cytokines cannot be excluded. Interestingly, inhibition of LD formation resulted in diverse patterns of inflammatory suppression under both stimulation conditions. It is conceivable that the process of LD formation differs when influenced by the two stimuli. For instance, treatment with LPS is known to result in a significant increase in cellular triglyceride levels in microglia [15]. Under the condition of OGD, it is possible that necrotic microglia release their triglycerides, while surviving cells exhibit a more robust autophagy capacity.

## 4. Materials and Methods

### 4.1. Isolation and Culture of Primary Microglia and Neurons

Cortices of neonatal C57BL/6 mice from postnatal day 0 to 1 were used to isolate primary microglia using a protocol with minor modifications [17]. The cortices were collected in Hank’s Balanced Salt Solution (HBSS) on ice after the pups’ heads were severed, followed by removing the meningeal layers and blood vessels. Pooled cortices were digested with 0.25% trypsin in a 37 °C water bath for 15 min. After that, a volume of 200 µL of 10 mg/mL DNase was added to digest the sticky DNA released from dead cells of three to four cortices. The cell suspension was centrifuged at 300× *g* for 5 min. Using a 1-mL pipet tip, the pellet was gently suspended in 5 mL of warm culture medium. The resulting cell suspension was put into a 15 mL tube and centrifuged at 300× *g* for 5 min. The resultant pellet was grown on Poly-d-lysine (PDL)-coated T75 flasks after being suspended in 25 mL of astrocyte complete media (DMEM supplemented with 10% fetal bovine serum (FBS) and 1% penicillin/streptomycin). The following day, non-adherent cell debris was removed by changing the culture medium. The latter was replaced every third day. A confluent cell layer of astrocytes would form at the bottom of the flask after 5–7 days, and microglia would develop on top of the astrocytic layer. By shaking the culture flasks for 1 h at 200 rpm in an orbital shaker at a constant 37 °C or 40 min at 0.05% moderate trypsinization, microglia were separated from mixed glial cell cultures. The microglia were cultured in the complete medium (DMEM/F12 (PAN-Biotech, Aidenbach, Germany) supplemented with 10% FBS and 1% Penicillin/Streptomycin). These cells were then passed three times to remove the mixed astrocytes inside.

Cortical neurons from E16.5 mouse embryos were procured and subsequently isolated. The isolated cortex pieces underwent trypsinization in a 37 °C water bath for 14 min, followed by dissociation through fire-polished Pasteur pipettes. These neurons were then cultured on 24-well plates coated with PDL at a density of 100,000 cells per square centimeter. The culture medium consisted of neurobasal medium (Gibco, Darmstadt, Germany) supplemented with HEPES (Sigma-Aldrich, St. Louis, MO, USA), transferrin (Sigma-Aldrich, St. Louis, MO, USA), penicillin/streptomycin, L-glutamine (Seromed, Dollnstein, Germany), and B27 supplement (Gibco, Darmstadt, Germany). The cells were utilized for 5 days in vitro.

### 4.2. Oxygen-Glucose Deprivation (OGD)

OGD was performed to generate hypoxic conditions in vitro. The seeding density of microglia was 40,000 cells per well for 24-well plates and 200,000 cells per well for 6-well plates. The given time for the cells to adhere was around 24 h. After confluency of 80 to 90% of cells, they were washed twice with phosphate-buffered saline (PBS) per well and received BSS0 solution (116 mmol/L NaCl, 5.4 mmol/L KCl, 0.8 mmol/L MgSO_4_, 1 mmol/L NaH2PO4H2O, 26.2 mmol/L NaHCO3, 10 mmol/L HEPES, 0.01 mmol/L glycine, and 1.8 mmol/L CaCl2, pH 7.2–7.4). Subsequently, the cells were put into the hypoxia chamber (<0.5% O_2_, 5% CO_2,_ and 95% N_2_ at 37 °C) and incubated for the designated time. At the end of hypoxia, the cells were taken out of the chamber, and the BSS0 was replaced by a cell culture medium, followed by incubation for 24 h at standard cell culture conditions. Cells incubated under standard cell culture conditions without hypoxia were defined as having 100% cell survival.

### 4.3. TGF-β1 siRNA Transfection of Microglia

As mentioned previously [17], a TGF-β1 small interfering RNA (siRNA) was used to knock down TGF-β1. Using Qiagen’s HiPerFect Transfection Reagent in Hilden, Germany, microglia were transfected with FlexiTube siRNA for mouse TGF-β1 (NM_011577). We selected the most optimal target sequence for RNA interference from the sequence provided by Qiagen (#SI00201684), maximizing efficiency. After 24 h of transfection, the cells were used for subsequent experiments. The details about the TGF-β1 mRNA and protein expression after treatment with different concentrations of TGF-β1 siRNA are indicated in our previous study [17]. In brief, using quantitative real-time polymerase chain reaction (qRT-PCR) and Western blot, quantitative analysis of TGF-β1 mRNA and protein expression in microglia at different concentrations of TGF-β1 siRNA groups (2 nM, 10 nM, and 50 nM) was measured. Around 50% of primary microglia were successfully transfected with 50 nM TGF-β1 siRNA. To confirm it, quantitative analysis of TGF-β1 expression in the 50 nM TGF-β1 siRNA by Western blot analysis normalized with the housekeeping protein GAPDH was conducted (Figure S1B,C).

### 4.4. Cell Viability

Using a previously reported methodology [37], the colorimetric MTT (Thiazolyl Blue Tetrazolium Bromide, Sigma-Aldrich, St. Louis, MO, USA) assay was used to measure cell viability. Briefly, this well-established colorimetric assay makes use of metabolically active cells, which convert yellow tetrazolium salt (3-(4,5-dimethylthiazol-2-yl)-2,5-diphenyltetrazolium bromide (MTT)) to purple formazan crystals. After OGD and reoxygenation, MTT at a concentration of 0.5 mg/mL was added to the cells and incubated for 3 h in an incubator at 37 °C. The fluid was aspirated to stop the colorimetric reaction, and dimethyl sulfoxide (DMSO) was then added in its place. Thereafter, a volume of 200 µL of the solubilized crystal solution was transferred into a 96-well microtiter plate, and the absorbance was measured at 570 nm on a Tecan Sunrise Microplate Reader. The results are expressed as a percentage of the control group.

### 4.5. Lipopolysaccharide (LPS) and Triacsin C Treatment

To induce LD formation, microglial cells were treated for 24 h with 2 µg/mL LPS in the microglia complete medium. Controls received vehicle solution (PBS) only. To inhibit LD formation, microglial cells were pretreated with 1 μM Triacsin C (Cayman Chemical, Ann Arbor, MI, USA) in microglia complete medium.

### 4.6. RNA Extraction and Real-Time qRT-PCR

Following the manufacturer’s instructions, total RNA was extracted using the Trizol reagent (Invitrogen, Waltham, CA, USA). qRT-PCR was carried out using the KAPA SYBR^®^ FAST One-Step Kit for LightCycler^®^480 (Merck Group, Darmstadt, Germany), and the PCR primers were bought from Eurofins Genomics (Ebersberg, Germany). More details about the primer information are attached in Table S1. Using the 2^−ΔΔCt^ method, all calculations for the findings of the relative expression analysis were standardized to PPIA.

### 4.7. Protein Extraction and Western Blot Analyses

Cells at 70–80% confluency were treated with the indicated conditions. To extract protein from the primary microglia of different experimental groups, the medium was aspirated, and cells were washed twice with PBS. Afterward, cells were gently agitated for 30 min while being scraped into a radioimmunoprecipitation lysis buffer from Thermo Fisher Scientific in Waltham, MA, USA. For 10 min, cell lysates were centrifuged at 12,000× *g*. The micro-bicinchoninic acid assay (Thermo Fisher Scientific, Waltham, MA, USA) was used to assess total protein quantities. Cell extracts were heated to 95 °C for 5 min while being treated with sample buffer (dithiothreitol, 0.1% SDS, 0.1 M Tris HCl, pH 7.0) before being separated on 10–12% SDS–polyacrylamide gel electrophoresis gels. After transfer on polyvinylidene fluoride membranes (Merck Group, Darmstadt, Germany), the membranes were successively blocked in 5% milk diluted in Tris-buffered saline solution with 1% Tween-20 for 1 h at room temperature, stained with primary antibodies overnight, and then incubated for 1 h with a secondary antibody linked to horseradish peroxidase. Protein signals were seen using the ChemiDocTM XRS+ imaging equipment from Bio-Rad (Hercules, CA, USA) after being submerged in an enhanced chemiluminescence reagent. Table S2 contains a list of the primary antibodies.

### 4.8. Immunocytochemistry Staining

Prior to being incubated with the primary antibody, the slides or wells were fixed with 4% paraformaldehyde (PFA) and blocked with 10% donkey serum (DS) and 1% bovine serum albumin (BSA) in PBS with Tween 20 (PBST). The indicated primary antibodies were then applied to the cell samples overnight at 4 °C in primary antibody diluent (Table S2). The samples were then subjected to the appropriate secondary antibody for 1 h at room temperature after being washed three times in PBS. In addition, DNA in cell nuclei was stained with 4, 6-diamidino-2-phenylindole (DAPI, 1:10,000; AppliChem, Darmstadt, Germany) for 10 min at room temperature. The National Institutes of Health’s ImageJ program was used to count cells and measure intensity.

### 4.9. Immunostaining for Lipid Droplets in Primary Microglia

The microglial cells were grown on the chamber slides. After treatment, they were briefly washed two times with ice-cold PBS and then fixed with 4% PFA for 15 min at room temperature. Subsequently, after brief washing in PBS, cells were permeabilized in PBS with 0.25% Triton X-100 at room temperature for 10 min. To reduce nonspecific binding, slides were blocked with 10% DS and 1% BSA in PBS with 0.1% TritonX-100 for 1 h followed by BODIPY staining. For BODIPY staining, the cells were stained with BODIPY 493/503 (D3922, 1 mg/mL, Thermo Fisher Scientific, Waltham, MA, USA) for 30 min at room temperature. Thereafter, microglia were washed three times with PBS for 5 min each wash and then counterstained with DAPI staining for 10 min at room temperature. The software ImageJ (National Institutes of Health) was used for cell counting and intensity quantification.

### 4.10. Statistical Analysis

For all data, the means and standard deviation are shown. For data processing and charting, GraphPad Prism 9.0 (GraphPad Software, San Diego, CA, USA) was used. The sample comparison between multiple groups was performed using a one-way analysis of variance (ANOVA), and the significance of the comparison between the two groups was evaluated using the t-test. Effect sizes were between 0.25 and 0.3. A *p*-value of 0.05 or less was considered statistically significant.

## 5. Conclusions

In summary, our findings underscore the role of TGF-β1 as a pivotal regulator of overall microglial inflammation and a potential factor in suppressing LD accumulation. In an in vitro model of stroke, both OGD and LPS stimulate LD accumulation. Yet, these two detrimental stimuli differentially modulate TGF-β1 expression. Elevated TGF-β1 concentrations significantly mitigate both inflammation and LD accumulation. Conversely, TGF-β1 knockdown enhances microglial cell inflammation and increases LD accumulation. The presence of Triacsin C prevents LD accumulation and promotes microglial polarization towards an anti-inflammatory profile. This modulation of TGF-β1 expression patterns and LD levels affects intercellular communication processes between microglia and neurons, particularly influencing neuronal viability. Therefore, the current results reveal, for the first time, that TGF-β1 serves a protective role against microglia-mediated neuroinflammation and inhibits LD accumulation. This suggests that this study may offer a novel perspective for stroke treatment. However, further investigation is needed to ascertain whether or not LD acts as a consequence of inflammation, a cause of inflammation, or if both factors mutually influence each other.

## Data Availability

Data contained within the article.

## Supplementary Materials

The following supporting information can be downloaded at: https://www.mdpi.com/article/10.3390/ijms242417329/s1.

## Abbreviations

ANOVA	Analysis of variance
BSA	Bovine serum albumin
CNS	Central nervous system
CD206	Cluster of Differentiation 206
DMSO	Dimethyl sulfoxide
DS	Donkey serum
FBS	Fetal bovine serum
HBSS	Hank’s Balanced Salt Solution
iNOS	inducible nitric oxide synthase
IL-1β	Interleukin-1β
LD	Lipid droplet
LPS	Lipopolysaccharide
MTT	3-(4,5-dimethylthiazol-2-yl)-2,5-diphenyltetrazolium bromide
OGD	Oxygen-glucose deprivation
PDL	Poly-d-lysine
PBS	Phosphate-buffered saline
PBST	PBS with Tween 20
PFA	Paraformaldehyde
PLIN2	Perilipin-2
qRT-PCR	quantitative real-time polymerase chain reaction
siRNA	small interfering RNA
TGF-β1	Transforming growth factor-β1
TNF-α	Tumor necrosis factor-α
