# Supplementary material for: TGF-β1 Decreases Microglia-Mediated Neuroinflammation and Lipid Droplet Accumulation in an In Vitro Stroke Model
Source: Int J Mol Sci. 2023 Dec 10;24(24):17329. doi: 10.3390/ijms242417329 (PMC10743979; doi:10.3390/ijms242417329)
Supplement: Supplementary file 1 [file ijms-24-17329-s001.zip › ijms-2712843-supplementary.pdf]

SUPPLEMENTARY MATERIALS

TGF-β1 Decreases Microglia-Mediated Neuroinflammation and Lipid Droplet Accumulation in an In Vitro Stroke Model

Wenqiang Xin[1], Yongli Pan[1], Wei Wei[1], Stefan T Gerner[2,3], Sabine Huber[2], Martin Juenemann[2], Marius Butz[2,4], Mathias Bähr[1], Hagen B Huttner[2], and Thorsten R Doeppner[1-3,5-6]

[1]Department of Neurology, University of Göttingen Medical School, Göttingen, Germany.

[2]Department of Neurology, University of Giessen Medical School, Giessen, Germany.

[3]Center for Mind, Brain and Behavior (CMBB), University of Marburg and Justus Liebig University Giessen, Germany.

[4]Heart and Brain Research Group, Kerckhoff Heart and Thorax Center, Bad Nauheim, Germany.

[5]Department of Anatomy and Cell Biology, Medical University of Varna, Varna, Bulgaria.

[6]Research Institute for Health Sciences and Technologies (SABITA), Medipol University, Istanbul, Turkey.

Correspondence:

Thorsten R. Doeppner, MD-M.Sc.

Department of Neurology

University of Giessen Medical School, Giessen, Germany

Phone: +49-641- 98545393

Email: thorsten.doeppner@neuro.med.uni-giessen.de

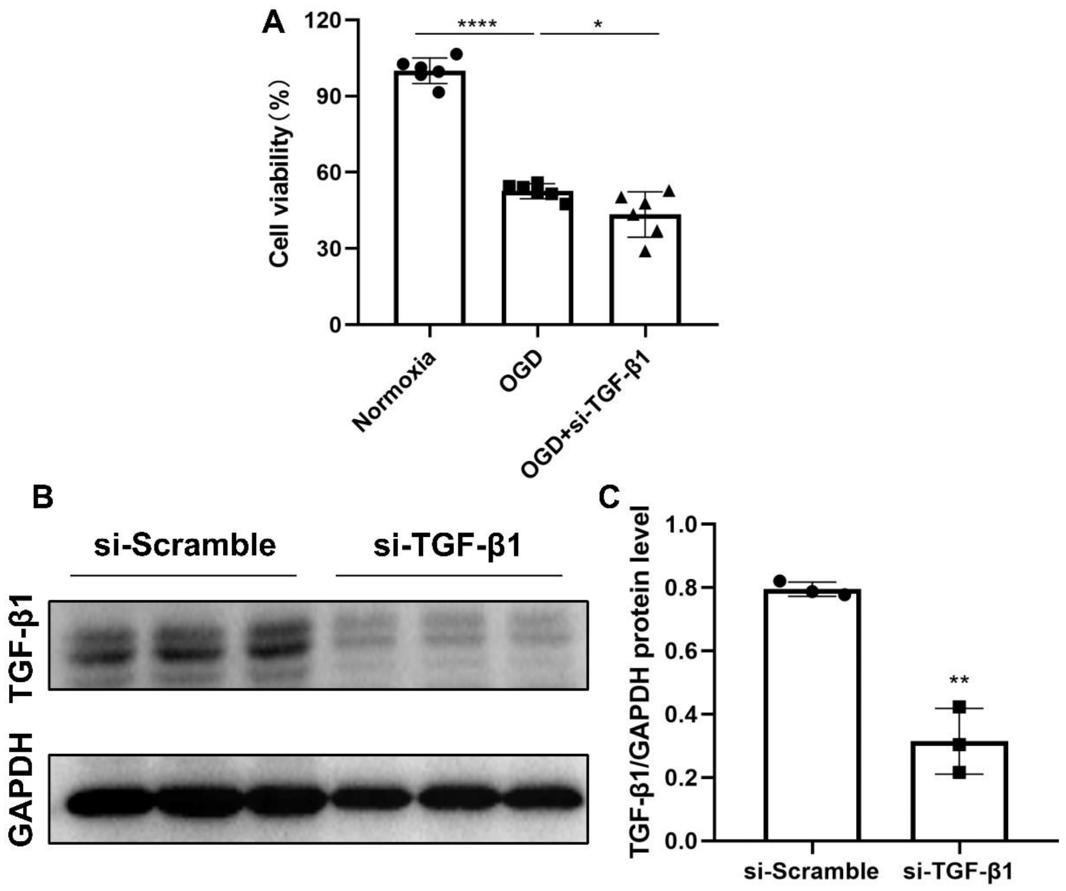

Figure S1. TGF-β1 siRNA successfully reduces both the expression of TGF-β1 and cell injury in microglia. (A) The MTT (Thiazolyl Blue Tetrazolium Bromide) assay was used to test microglial cell viability after OGD treated with or without TGF-β1 siRNA. Cells incubated under standard cell culture conditions ('Normoxia') were defined as 100 % cell survival (n = 6). (B-C) A quantitative analysis of TGF-β1 expression using 50 nM of TGF-β1 siRNA for 24 h by western blot analysis normalized with the housekeeping protein GAPDH was conducted in primary microglia. *p < 0.05; **p < 0.01; ****p < 0.0001. OGD, oxygen-glucose-deprivation; si-TGF-β1, transforming growth factor-β1 siRNA; si-Scramble; siRNA scramble.

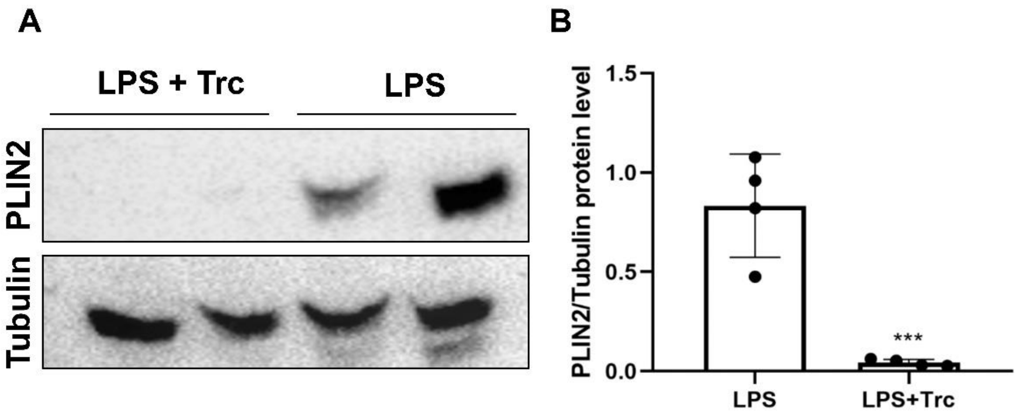

Figure S2. The effect of Triacsin C on LPS-induced LD accumulation in primary microglia. Western blot analysis depicts the expression of PLIN2 protein in primary microglia under different conditions, including LPS-treated cells and LPS-treated cells with Triacsin C (n = 4). ***$p < 0.001$. LD, lipid droplet(s); LPS, lipopolysaccharide; PLIN2, perilipin2; Trc, Triacsin C.

Table S1. Sequence information of quantitative real-time PCR analysis primers.

Gene name	Primer sequence (5′-3′)
**rCD206 F**	CTCTGTTCAGCTATTGGACGC
**rCD206 R**	CGGAATTTCTGGGATTCAGCTTC
**riNOS F**	AGGAACCTACCAGCTCACTCTG
**riNOS R**	TTTCCTGTGCTGTGCTACAGTT
**rIL-1β F**	GCAACTGTTCCTGAACTCAACT
**rIL-1β R**	ATCTTTTGGGGTCCGTCCAACT
**rIL-10 F**	AGAAAAGAGAGCTCCATCATGC
**rIL-10 R**	TTATTGTCTTCCCGGCTGTACT
**rTNF-α F**	AAGCCTGTAGCCCACGTCGTA
**rTNF-α R**	GGCACCACTAGTTGGTTGTCTTTG
**rPLIN2 F**	ACACCCTCCTGTCCAACATC
**rPLIN2 R**	AAGGGACCTACCAGCCAGTT
**rTGF-β1 F**	CCAGATCCTGTCCAAACTAAGG
**rTGF-β1 R**	CTCTTTAGCATAGTAGTCCGCT
**rPPIA F**	GAGCTGTTTGCAGACAAAGTTC
**rPPIA R**	CCCTGGCACATGAATCCTGG

Table S2. Antibodies used for immunofluorescence staining and western blots.

Antibody	Concentration	Supplier	Cat. No.	Species
**Antibody information for immunofluorescence staining**				
Anti-CD68	1:250	BioRad	MCA341F	rat
Anti-Iba1	1:250	WAKO	011-27911	rabbit
Anti-CD11b	1:250	Abcam	ab75476	rabbit
Anti-CX3CR1	1:250	Thermo Fisher Scientific	PA5-19910	rabbit
**Antibody information for western blots**				
Anti-TGF-β1	1:1,000	Abcam	ab92486	rabbit
Anti-PLIN2	1:1,000	PROGEN	GP42	Guinea pig
Anti-Tubulin	1:10,000	GeneTex	GTX628802	mouse
Anti-GAPDH	1:10,000	GeneTex	GTX627408	mouse
Anti-rabbit	1:10,000	Abcam	ab97051	goat
Anti-mouse	1:10,000	Abcam	ab97023	goat
Anti-Guinea pig	1:10,000	Abcam	ab6908	goat

Supplementary full scans of western blots

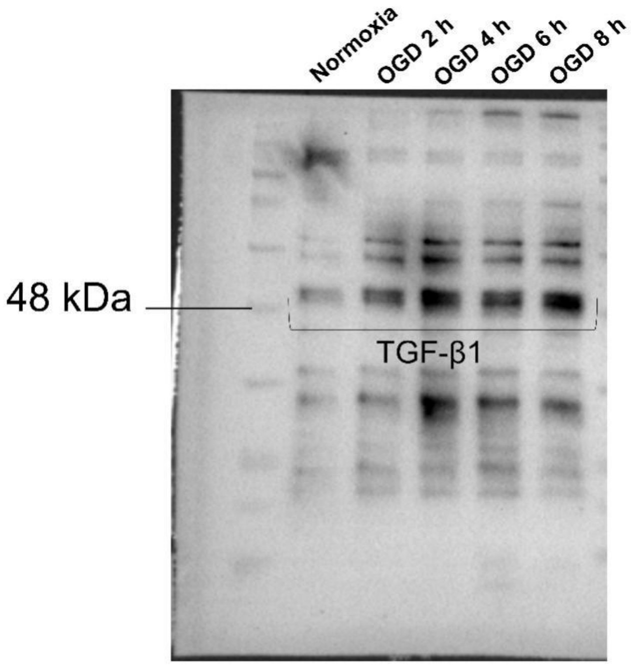

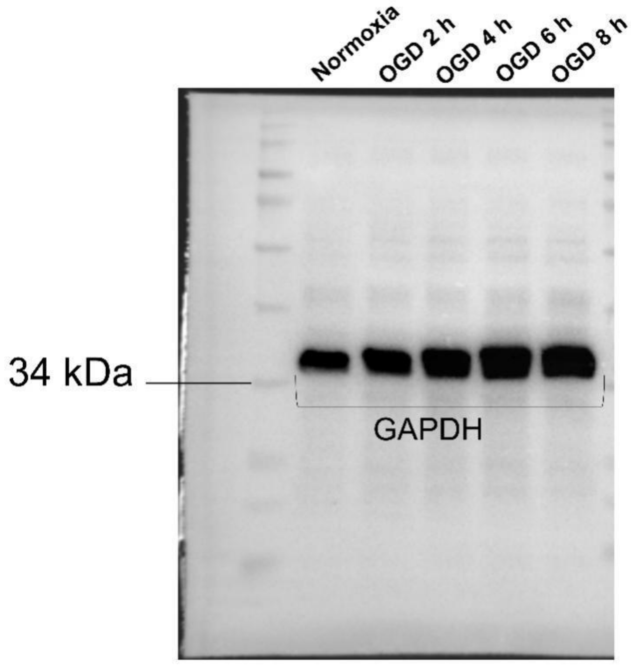

Full scans of western blots shown in Fig. 1G. TGF-β1 and GAPDH.

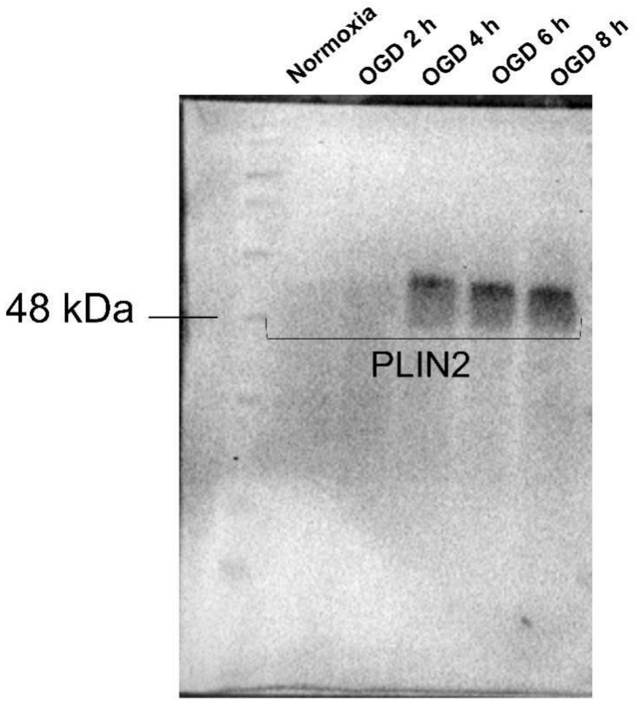

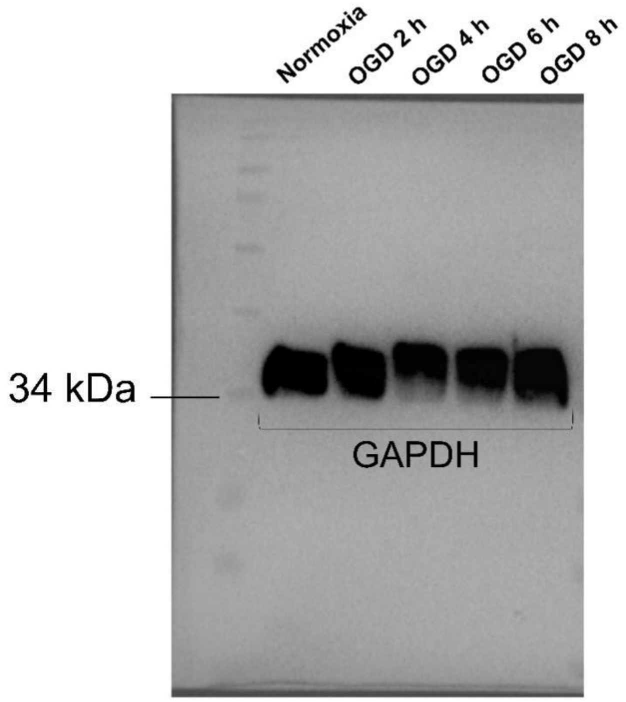

Full scans of western blots shown in Fig. 1H. PLIN2 and GAPDH.

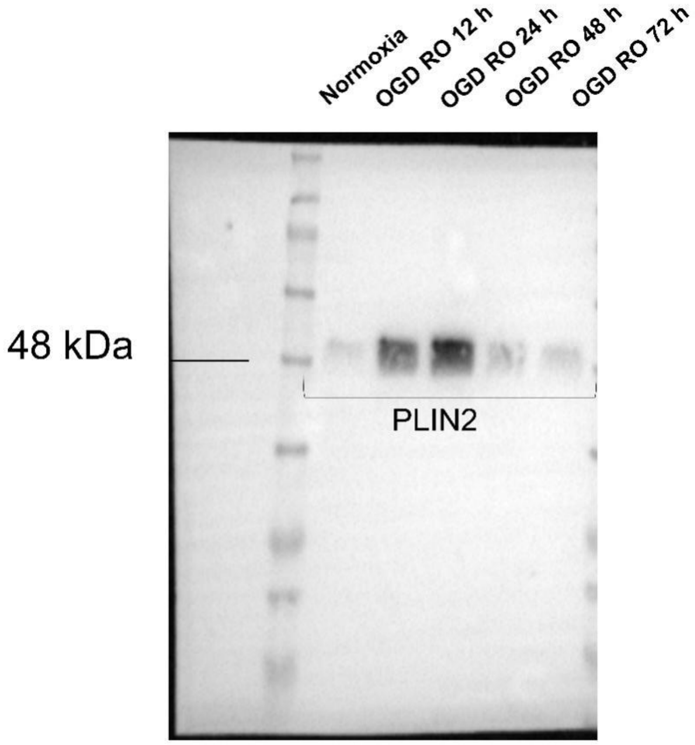

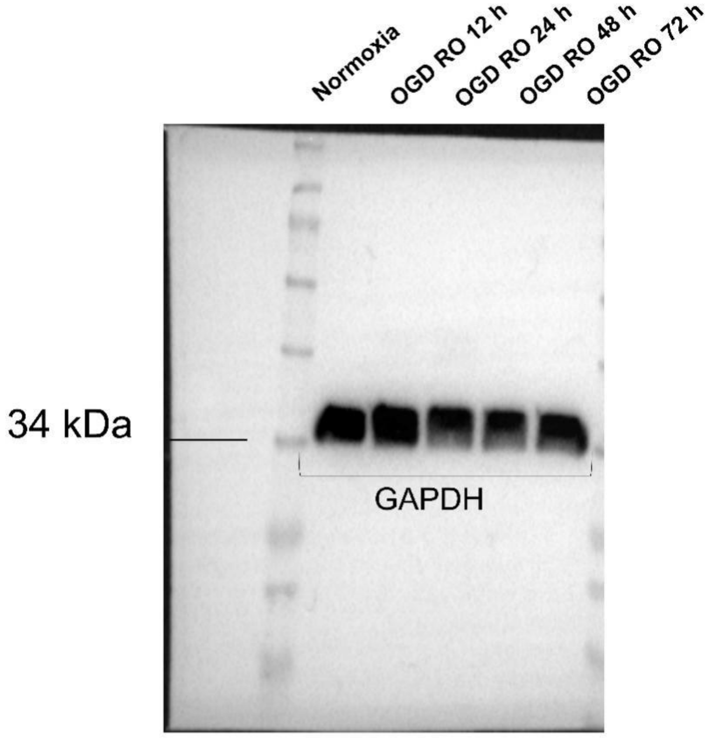

Full scans of western blots shown in Fig. 2D. PLIN2 and GAPDH.

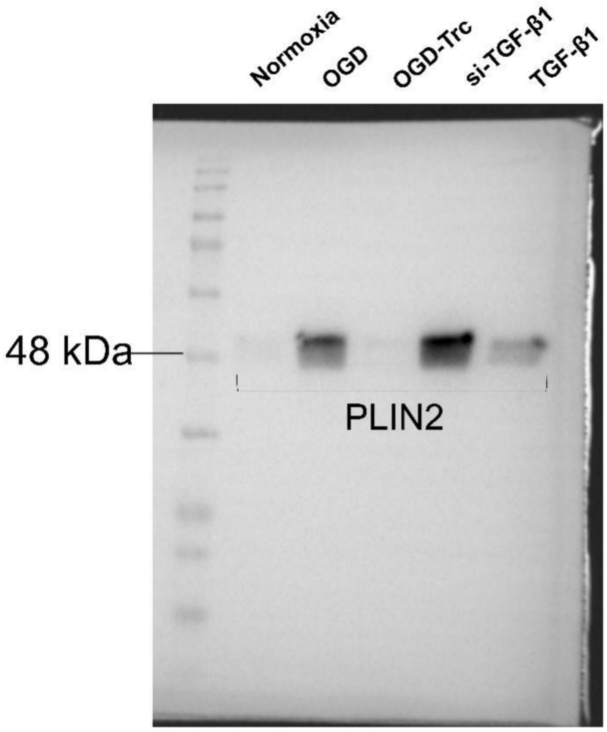

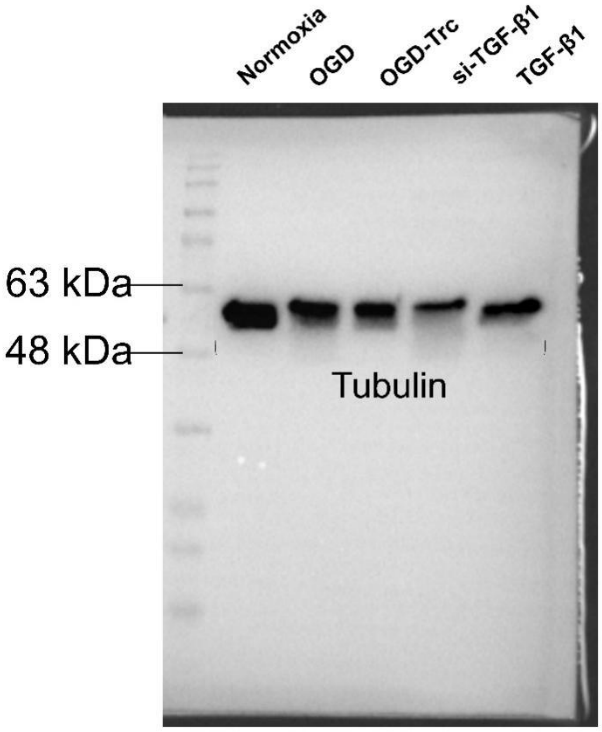

Full scans of western blots shown in Fig. 3F. PLIN2 and Tubulin.

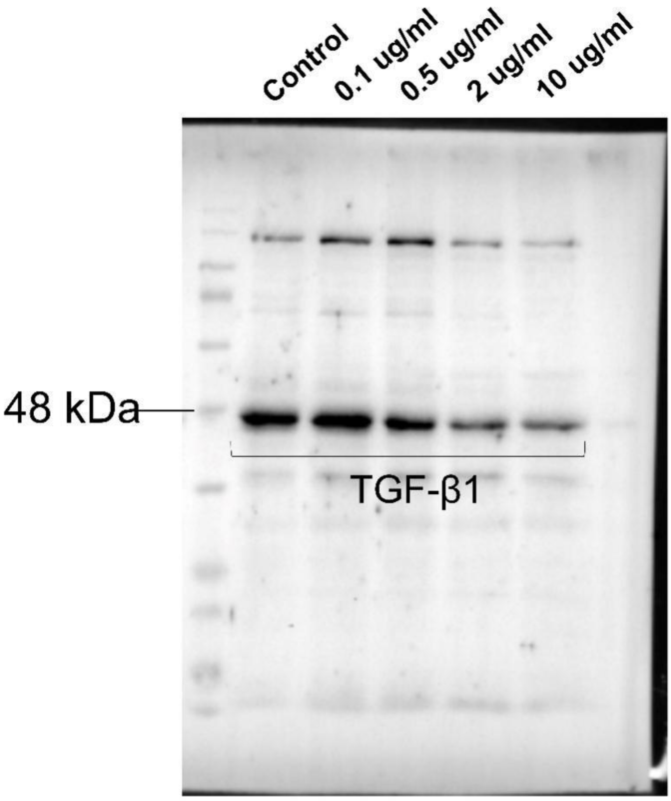

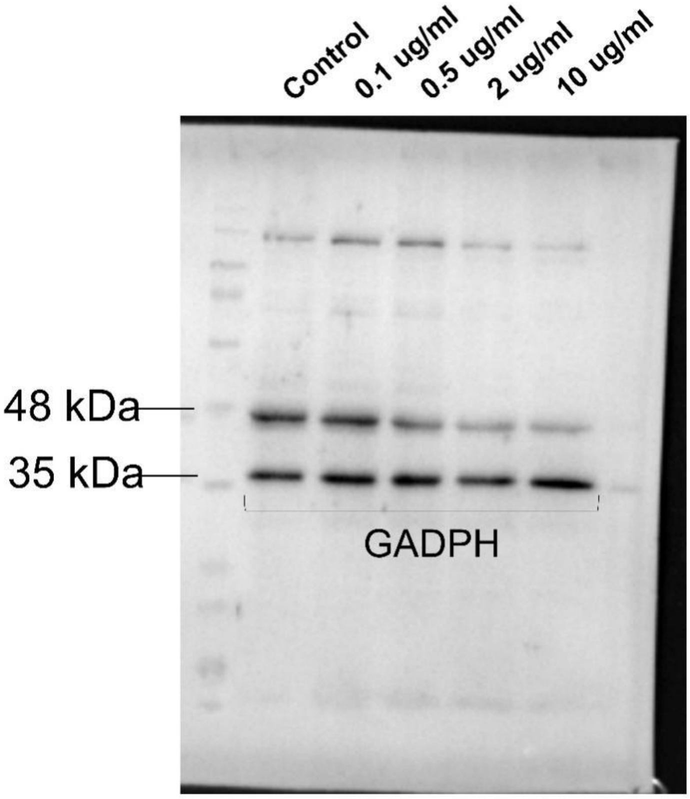

Full scans of western blots shown in Fig. 4A. TGF-β1 and GAPDH.

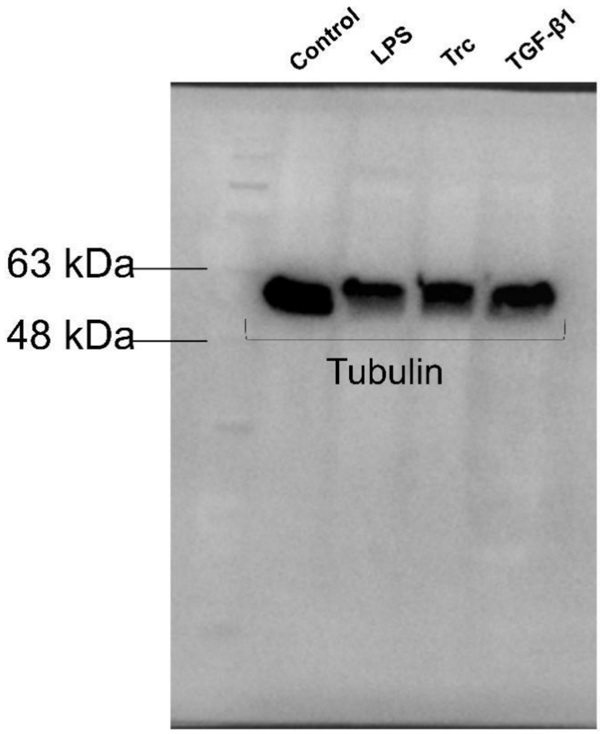

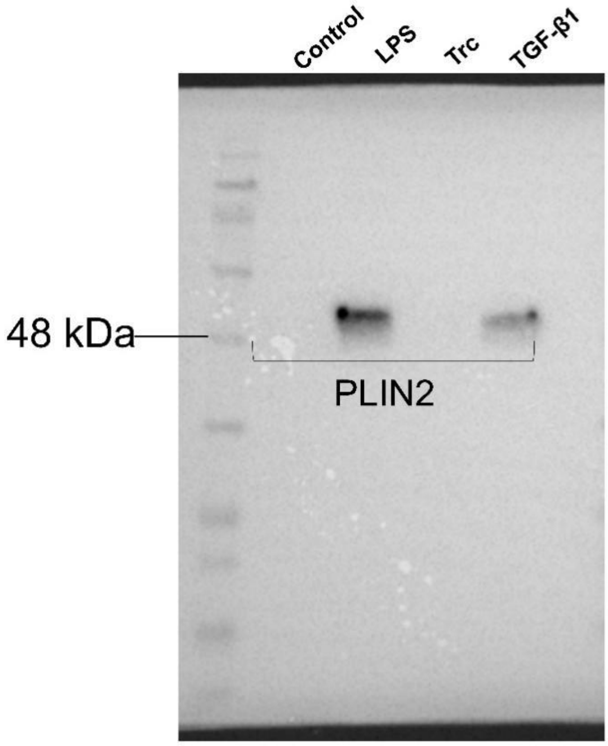

Full scans of western blots shown in Fig. 4C. PLIN2 and Tubulin.

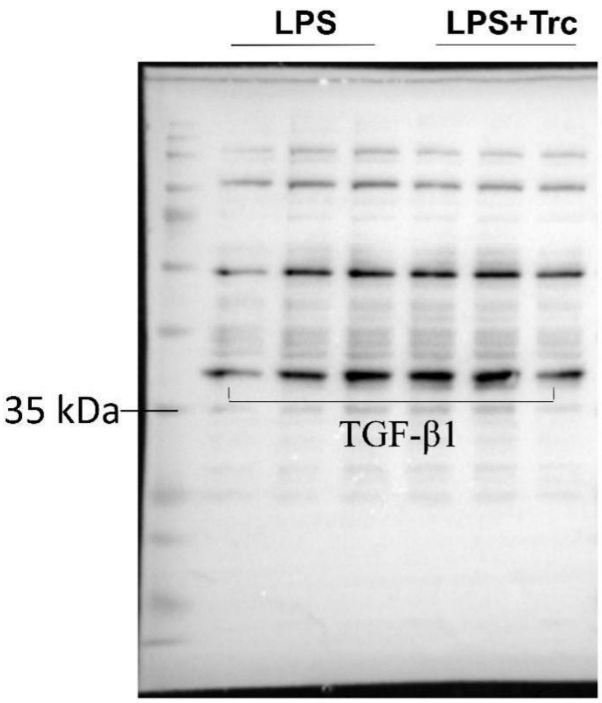

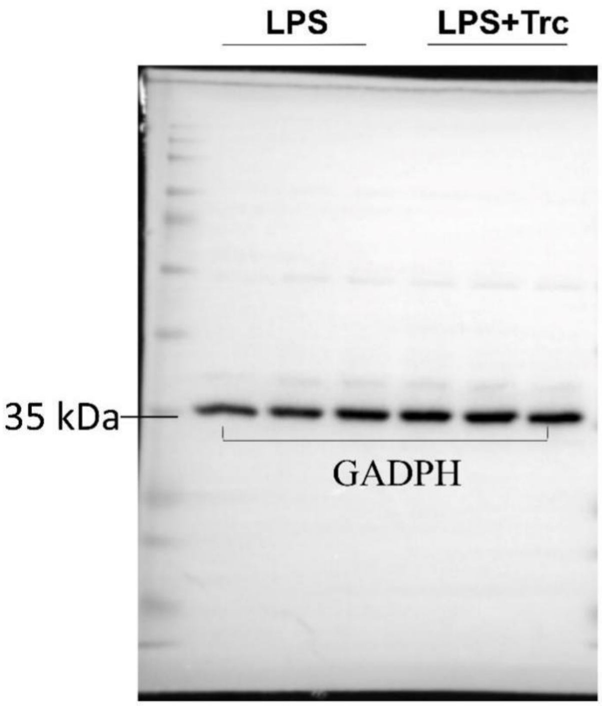

Full scans of western blots shown in Fig. 6M. TGF-β1 and GAPDH.

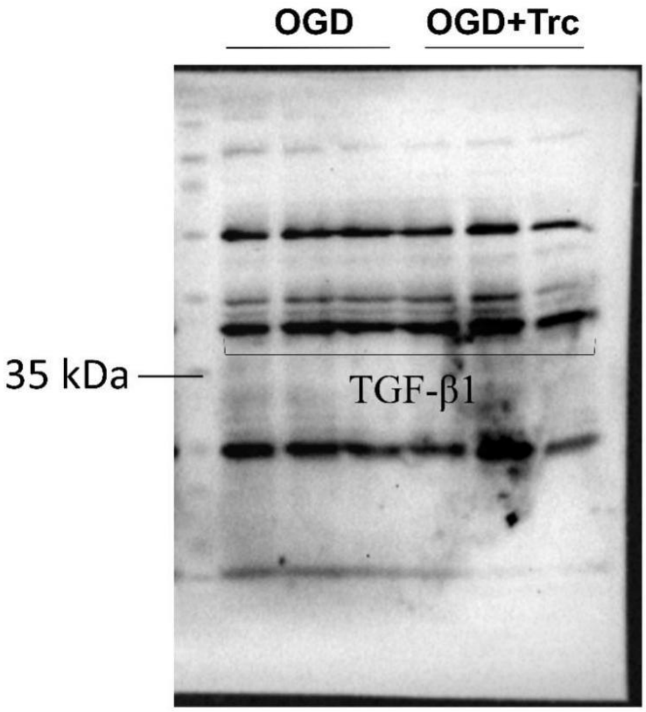

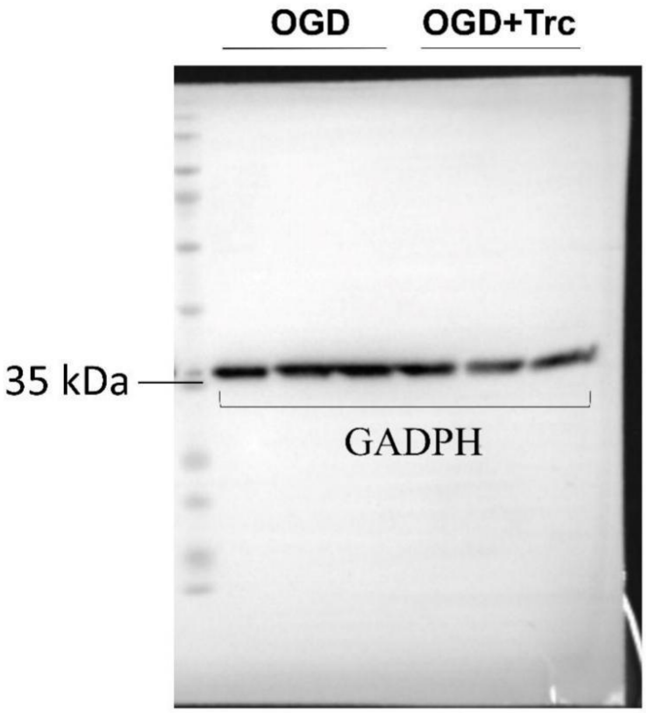

Full scans of western blots shown in Fig. 6N. TGF-β1 and GAPDH.

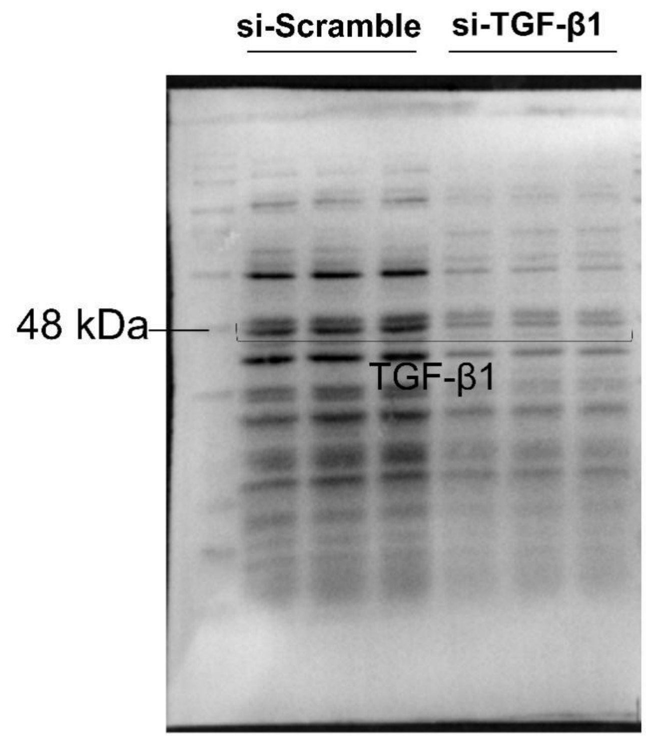

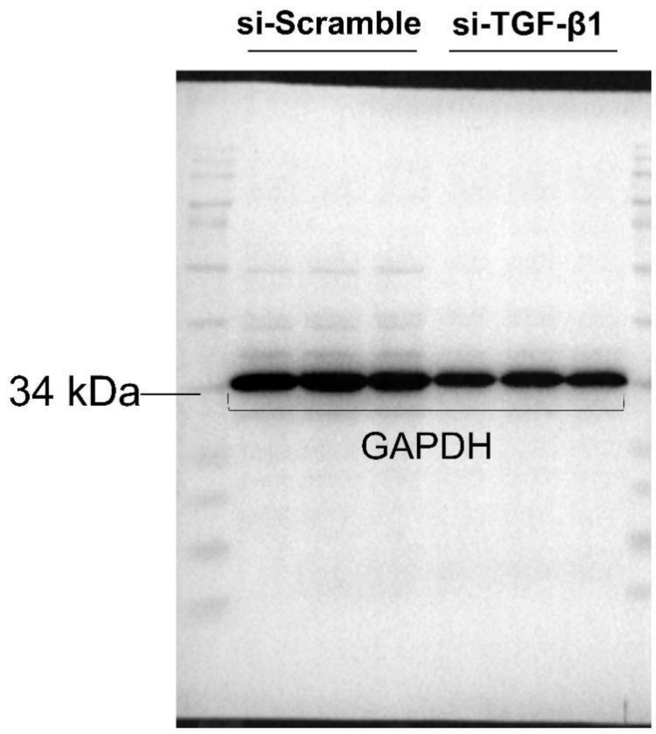

Full scans of western blots shown in Fig. S1. TGF-β1 and GAPDH.

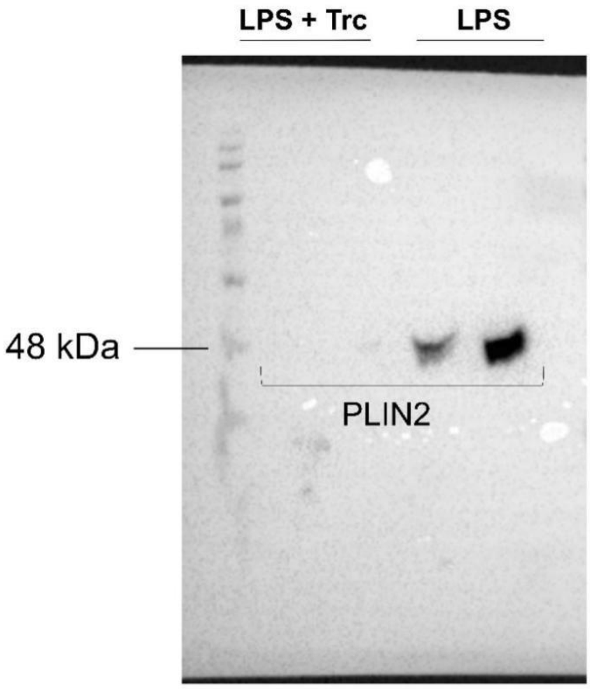

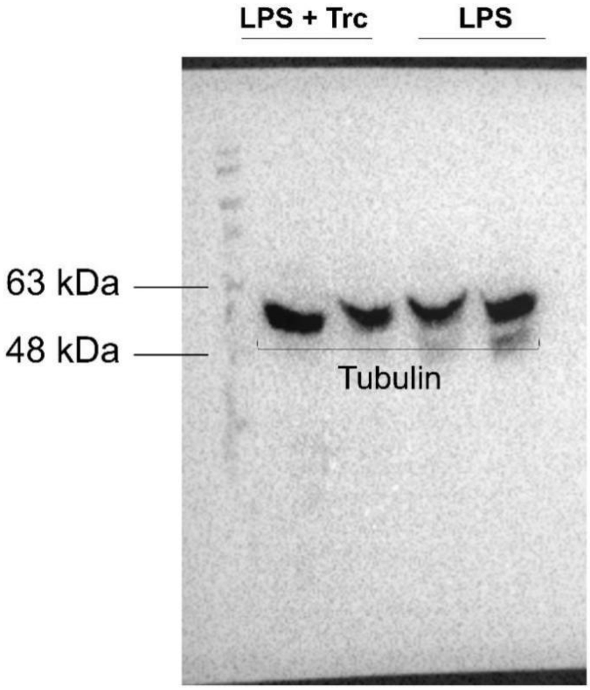

Full scans of western blots shown in Fig. S4. PLIN2 and Tubulin.

Supplementary negative staining controls in primary microglia

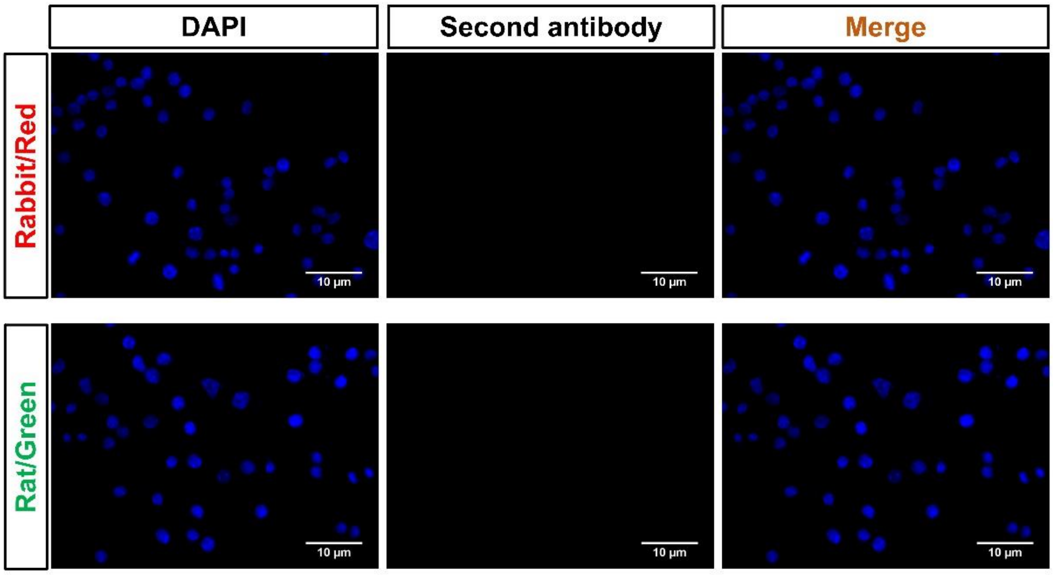

Upper panel: As a negative control, the secondary antibody Cy3 donkey anti-rabbit IgG (1:250, red) was incubated with primary microglia, and there is no unspecific staining on the slides.

Lower panel: As a negative control, the secondary antibody Alexa 488 donkey anti-rat IgG (1:250, green) was incubated with primary microglia, and there is no unspecific staining on the slides.
